# A Ziegler-type spherical cap model reveals early stage ethylene polymerization growth versus catalyst fragmentation relationships

**DOI:** 10.1038/s41467-022-32635-z

**Published:** 2022-08-24

**Authors:** Koen W. Bossers, Laurens D. B. Mandemaker, Nikolaos Nikolopoulos, Yuanshuai Liu, Marcus Rohnke, Peter de Peinder, Bas J. P. Terlingen, Felix Walther, Joren M. Dorresteijn, Thomas Hartman, Bert M. Weckhuysen

**Affiliations:** 1 Inorganic Chemistry & Catalysis, Debye Institute for Nanomaterials Science, Utrecht University, Utrecht, The Netherlands; 2 Qingdao Institute of Bioenergy and Bioprocess Technology, Chinese Academy of Sciences, Qingdao, China; 3 Center for Materials Research, Justus Liebig University, Giessen, Germany; 4 Institute of Physical Chemistry, Justus Liebig University, Giessen, Germany; 5 VibSpec, Haaftenlaan 28, Tiel, The Netherlands

**Keywords:** Heterogeneous catalysis, Reaction kinetics and dynamics, Catalytic mechanisms

## Abstract

Polyolefin catalysts are characterized by their hierarchically complex nature, which complicates studies on the interplay between the catalyst and formed polymer phases. Here, the missing link in the morphology gap between planar model systems and industrially relevant spherical catalyst particles is introduced through the use of a spherical cap Ziegler-type catalyst model system for the polymerization of ethylene. More specifically, a moisture-stable LaOCl framework with enhanced imaging contrast has been designed to support the TiCl_4_ pre-active site, which could mimic the behaviour of the highly hygroscopic and industrially used MgCl_2_ framework. As a function of polymerization time, the fragmentation behaviour of the LaOCl framework changed from a mixture of the shrinking core (i.e., peeling off small polyethylene fragments at the surface) and continuous bisection (i.e., internal cleavage of the framework) into dominantly a continuous bisection model, which is linked to the evolution of the estimated polyethylene volume and the fraction of crystalline polyethylene formed. The combination of the spherical cap model system and the used advanced micro-spectroscopy toolbox, opens the route for high-throughput screening of catalyst functions with industrially relevant morphologies on the nano-scale.

## Abstract (web summary)

Ziegler-type polyolefin catalysts have proven to be hard to characterize. Here the authors present a model system consisting of patterned LaOCl spherical caps, simulating bulk particles while facilitating the use of micro(-spectro)scopic characterization techniques specifically aimed at surfaces.

## Introduction

The Ziegler-type (e.g., Ziegler and Ziegler-Natta) catalysts discovered in the 1950’s by Karl Ziegler and Giulio Natta are the dominant α-olefin polymerization catalysts for the production of different polyethylene grades, such as high-density polyethylene (HDPE) and isotactic polypropylene (i-PP)^1^. The current generation Ziegler-type catalysts are based on the chemisorption of a TiCl_4_ pre-active site species on an activated MgCl_2_ support matrix, followed by the reduction and alkylation with a trialkylaluminium co-catalyst^2^. In the case of propylene polymerization, additional Lewis base molecules are added either during the synthesis, which are called internal donors, or the polymerization reaction, which are called external donors, to provide local stereoregular control for the production of highly isotactic polypropylene^3^.

Ziegler-type catalysts are both hierarchically complex and highly sensitive towards polar compounds, such as O_2_ and H_2_O, that complicate catalyst structure, polymerization activity and polymer properties studies^4,5^. Nonetheless, both theoretical, spectroscopic and high-throughput studies have given invaluable insights in the working mechanisms of Ziegler-type catalysts such as the nature of the exposed and unsaturated lattices on MgCl_2_ that can chemisorb TiCl_4_ and their relationship towards the formation of isotactic polypropylene^6–9^. However, an alternative strategy is to simplify the hierarchically complex nature of these catalysts altogether through the design of planar model systems^10^. These model systems have the additional advantage of being compatible with surface-sensitive spectroscopy and microscopy techniques, such as atomic force microscopy (AFM) and X-ray photoelectron spectroscopy (XPS)^11^. Somorjai and his group pioneered the field of using surface science techniques on planar model systems of Ziegler-type catalysts^12–16^. In one of their early works, they found experimental evidence for the TiCl_4_ activation mechanism as proposed theoretically by Arlman and Cossee using an ultra-thin MgCl_2_ film on a gold substrate^16,17^. Siokou and Ntais switched towards the use of a facile spin-coating technique to produce a Ziegler-type planar model system based on tetrahydrofuran (THF) adducts with MgCl_2_ and TiCl_4_, which is in close analogue to the chemical activation routes of MgCl_2_ used industrially^18–22^. The effect of the type of internal donor on the stabilization of the unsaturated (110) and (104) lattices for a Ziegler-Natta planar model system was nicely studied by the group of Niemantsverdriet by growing well-defined crystals on a SiO_2_/Si(100) substrate using a combined spin-coated and solvent vapour annealing approach^23–26^.

For these conventional MgCl_2_ based Ziegler-type catalyst model systems however, technical and experimental limitations are imposed due to the high moisture sensitivity of MgCl_2_, which will lead to morphology changes under ambient conditions. Recently, Piovano et al., reported a chlorinated δ-Al_2_O_3_ support matrix instead of MgCl_2_ to facilitate in-situ ethylene oligomerization at Al^3+^ sites for the production of a linear low-density polyethylene grade (LLDPE)^27,28^. The use of such non-conventional support matrixes for Ziegler-type catalysis inspired us to design a model system for ethylene polymerization using a LaOCl support matrix due to two reasons. First of all, LaOCl provides strong imaging contrast due to the high atomic weight of the lanthanide and, secondly, exceptional moisture stability to facilitate ambient measuring conditions for the selected advanced micro-spectroscopy toolbox. Whereas LaOCl has been reported as a promising catalyst material for the selective alkane activation, such as the conversion of methane to methyl chloride and ethane to vinyl chloride as well as the destruction of chlorinated hydrocarbons, it has not been reported yet to the best of our knowledge in the field of α-olefin polymerization^29–35^. Additionally, to bridge the gap between planar model systems and the industrially relevant highly spherical catalyst particles, a spherical cap model system is introduced here to the field of catalytic α-olefin polymerization. This LaOCl-based spherical cap model system will be studied with a versatile micro-spectroscopy toolbox consisting of photo-induced force microscopy (PiFM), which provides infrared (IR) nano-spectroscopy with the spatial resolution and additional topological information of AFM (AFM-IR)^36^, Raman microscopy, focused ion beam scanning electron microscopy energy dispersive X-ray spectroscopy (FIB-SEM-EDX), XPS and time-of-flight secondary ion mass spectrometry (ToF-SIMS). Additionally, a MgO/MgCl_2_ core-shell reference cap system, which showed high sensitivity to moisture under the ambient measuring conditions, was also prepared to compare polymerization activity and fragmentation behaviour of both types of support matrixes. By doing so, we were able to follow the interplay between the formation of polyethylene and fragmentation of the catalyst support matrixes with high imaging contrast and under ambient ex-situ measuring conditions at different ethylene polymerization times. Both fundamental fragmentation models, namely the shrinking core and continuous bisection, were occurring simultaneously at early polymerization times, and upon sufficient fragmentation of the LaOCl framework to overcome initial mass transfer limitations, switched towards a dominating continuous bisection behaviour. For the MgO/MgCl_2_ reference system, despite a weaker Z-contrast for chemical imaging and high moisture sensitivity, a higher ethylene polymerization activity was observed that was linked to a dominant continuous bisection fragmentation model even at the earliest polymerization yields, showing the high friability of the support matrix.

## Results and discussion

### Fabrication of the spherical cap model system

**Fig. 1:**
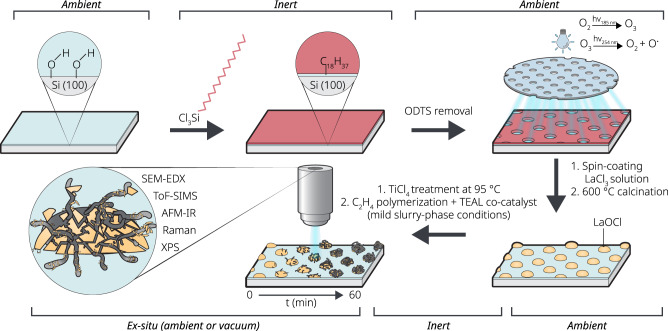
Schematic representation of the synthesis route of LaOCl spherical caps on a surface modified Si(100) wafer. Chemical modification of the Si(100) wafer surface, to generate hydrophilic circular patches (about 40 μm in diameter) separated by hydrophobic bars, is achieved through sequential grafting of the hydrophobic octadecyltrichlorosilane (ODTS) on a hydroxylated surface and selective etching with a photomask and UV/O_3_. Subsequent spin-coating and calcination of a LaCl_3 *_ 7 H_2_O solution in ethanol leads to the formation of LaOCl spherical caps exclusively inside the hydrophilic regions. After grafting of TiCl_4_ on the LaOCl spherical caps, ethylene polymerization was performed in slurry phase under mild conditions with a triethylaluminium co-catalyst at different reaction times. A toolbox, consisting of advanced micro-and-spectroscopic techniques, was used to follow the ethylene polymerization process ex-situ.

To bridge the morphology gap between conventional planar film model systems and the industrially relevant spherical catalyst particles, a combined photomasking and spin-coating technique was used for the synthesis of spherical caps on a suitable substrate, such as Si(100). An outline of these synthesis steps is given in Fig. 1, starting from the hydroxylation of the Si(100) wafer towards the grafting of an octadecyltrichlorosilane (ODTS) self-assembled monolayer (SAM). This SAM is then selectively etched away using a large area (20 × 20 mm^2^) TEM copper grid photo-mask with UV/ozone, followed by the spin-coating and calcination of the hydrated LaCl_3_ precursor salt to finally yield the LaOCl spherical caps. The selective etching of surface-anchored SAMs and subsequent coating with different metal oxides was demonstrated for the fabrication of flat square patches of TiO_2_ by Masuda et al., growth of zeolite domains by Ha et al., and the synthesis of well-defined KCl single crystals by van Delft et al.^37–39^. In this work, a LaOCl phase is chosen over a LaCl_3_ phase to support the Ti^3+^ active site due to the high stability of LaOCl against hydration which facilitates ambient measuring conditions when applicable, whereas LaCl_3_ is highly hygroscopic analogous to the conventional MgCl_2_ support matrix. It should be noted that LaOCl has a tetragonal crystal structure, whereas the conventional δ-MgCl_2_ phase, which resembles the δ-TiCl_3_ phase, has a hexagonal crystal structure. Additionally, other factors that explain the success story of MgCl_2_ as a support matrix are the similar ionic radii of Mg^2+^ (0.72 Å) and Ti^3+^ (0.67 Å) and Ti^4+^ (∼0.61 Å) as well as the similar bond lengths of Mg-Cl and Ti-Cl (respectively, Å and Å)^2^. In the case of La^3+^ both a larger ionic radius (∼1.22 Å) and longer La-Cl bond length (∼2.4 Å) is observed^40,41^. However, both MgCl_2_ and LaOCl are built up of successive layers of M^x+^ and Cl^−^ anions (in the case of LaOCl, LaO^+^ layers alternating with double Cl^−^ layers), where primary particles of these compounds are platelets loosely bound together through ionic interactions^42–44^. After grafting the TiCl_4_ active site precursor on the LaOCl spherical caps followed by the activation with a triethylaluminium (TEAL) co-catalyst and ethylene polymerization under mild conditions, e.g., 2 bar ethylene, slurry-phase at room temperature, the polyethylene-LaOCl composite spherical caps are studied in detail using a toolbox consisting of both surface-sensitive (micro)-spectroscopic and conventional micro-spectroscopic techniques.

Both the surface-sensitive techniques, XPS and ToF-SIMS, and the bulk material probing Raman microscopy on the as-synthesized LaOCl spherical caps confirms the formation of the LaOCl phase as shown in the Supplementary Information sections 1 and 2. Additionally, XPS shows that after coordination of TiCl_4_ to the LaOCl surface a minor amount of reduced Ti^3+^ are observed even before any contact with the reducing triethylaluminium (TEAL) co-catalyst. Although the majority of the Ti^x+^ species remain in the +4 oxidation state, it shows a possible similarity with the interaction and subsequent decomposition of CCl_4_ on a LaOCl catalyst material through CCl_3_^δ+^-Cl^δ−^ intermediate species as reported by our group^33,34^. The contribution of these minorly present reduced Ti^3+^ species to the overall catalytic activity after contact with a trialkylaluminium co-catalyst is under active spectroscopic investigation on a powdered-form LaOCl support matrix.

### Introducing the micro-spectroscopy toolbox

**Fig. 2:**
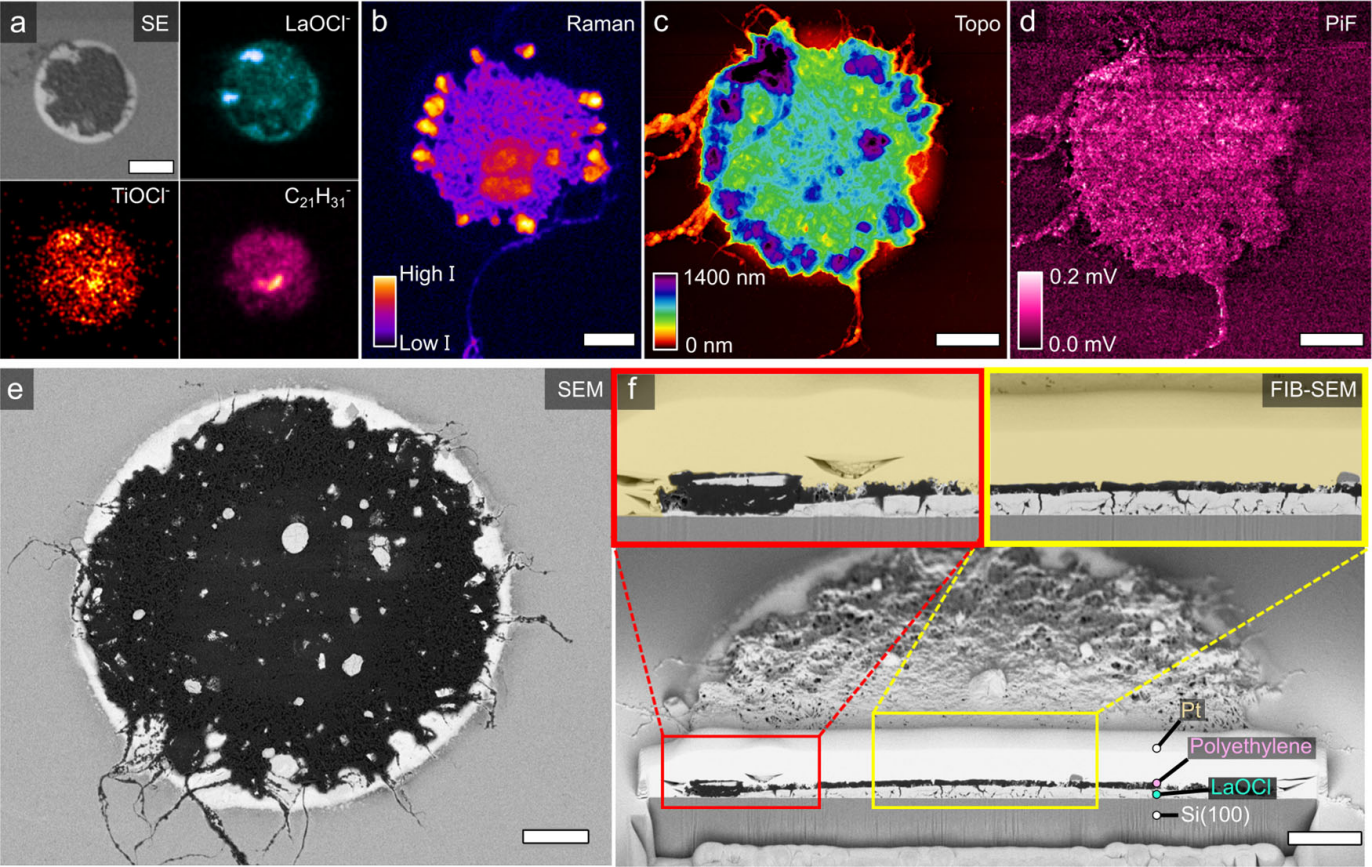
An overview of the micro-spectroscopic toolbox utilized in this work to study the interplay between the formed polyethylene and the LaOCl spherical cap model system demonstrated after 20 min of ethylene polymerization. **a** Time-of-flight secondary ion mass spectrometry (ToF-SIMS) that provides a distribution of the negatively charged mass species present on the surface, such as the selected LaOCl^−^, TiOCl^−^ and polyethylene characteristic C_21_H_31_^−^ fragments. **b** Raman microscopy map of the 2700–3100 cm^−1^ region shows the -CH stretching modes of polyethylene on a different LaOCl spherical cap. **c**, **d** Combined photo-induced force microscopy (PiFM) results that give both topological and morphological information in **c** and distribution of the summed δ(CH_2_) peaks at respectively 1461 cm^−1^ (*B*_1u_) and 1471 cm^−1^ (*B*_2u_) of the IR spectra in **d** of the same spherical cap. **e** Top-view scanning electron microscopy (SEM) image showing backscattered electrons that provides *Z*-contrast. In dark grey, polyethylene fibers are observed to grow from the LaOCl spherical cap (in bright white). **f** Focused ion beam (FIB)-SEM image of the same spherical cap, which shows a cross-section of the internal structure of the LaOCl spherical cap and the formed polyethylene phase. Zoom-ins are given in the red and yellow insets. All scale bars represent a width of 5 µm. A yellow, transparent overlay is provided that indicates the coated Pt layer, all though in some cases it is observed to penetrate into the porous polyethylene layer.

**Fig. 3:**
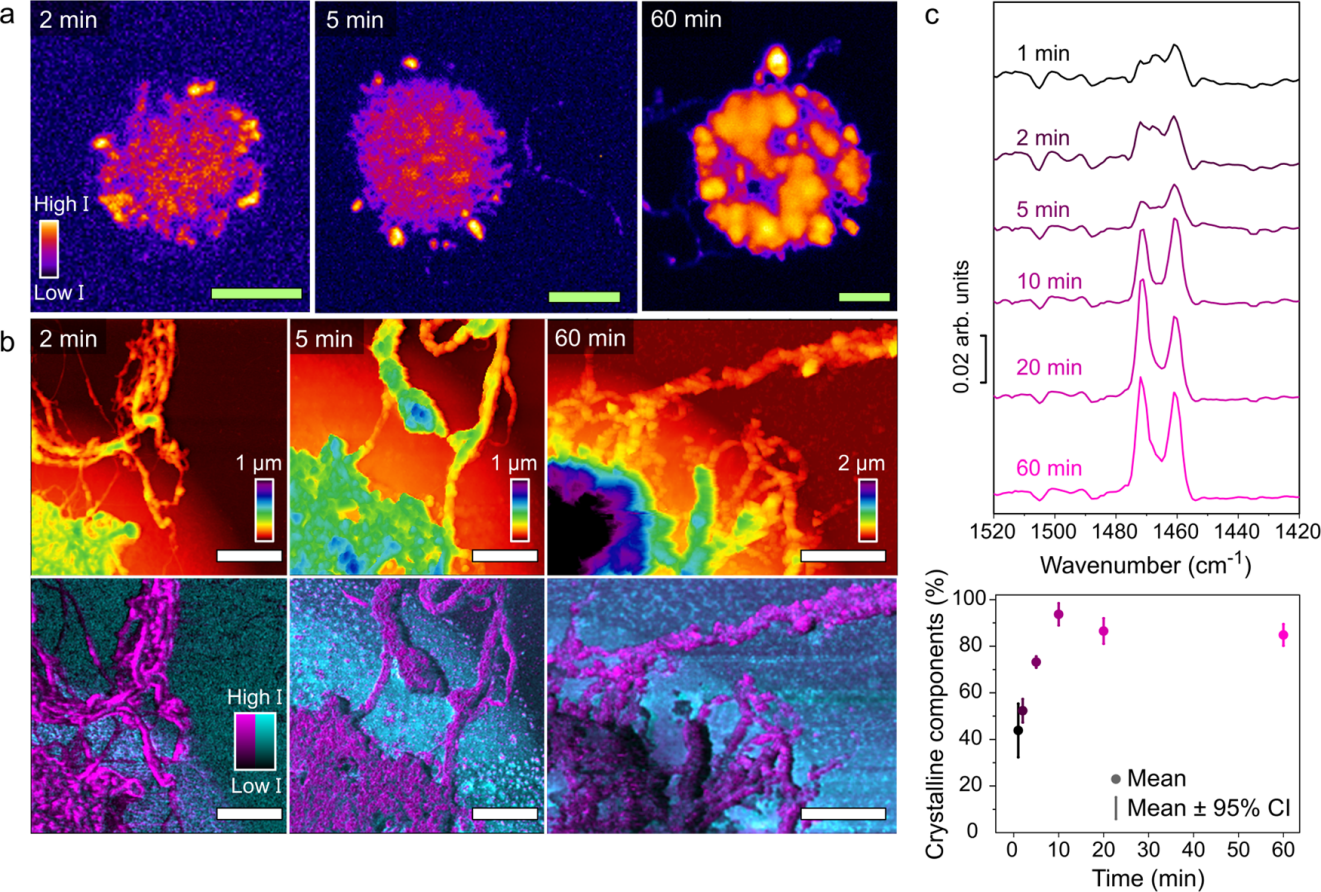
Raman microscopy and photo-induced force microscopy (PiFM) to study the crystallization of the formed polyethylene after 2, 5 and 60 min of ethylene polymerization. **a** Raman microscopy maps of the asymmetric and symmetric -CH_2_- stretching vibrations in the 2700–3100 cm^−1^ region. **b** The topological (top) and PiFM (bottom) images of zoom-ins on the spherical caps. The PiF images show the distribution of the -CH_2_- symmetric bending mode peaks in magenta and LaOCl surface adsorbed carbonate species acting as markers for LaOCl in cyan. The green (Raman microscopy) and white (PiFM topology) scale bars represent a width of respectively, 10 µm and 2 µm. **c** The photo-induced force (PiF) spectra that give the -CH_2_- symmetric bending mode peaks of crystalline polyethylene in an orthorhombic phase are shown after different polymerization times, averaged of 18 spectra measured on 9 different patterned catalysts per time. Multivariate curve resolution (MCR) analysis was performed to fit the individual spectra on 4 different spectral components. The fraction of the two crystalline components (1461 cm^−1^ and 1471 cm^−1^ bands) versus the single amorphous component (broad band at 1463 cm^−1^) is plotted in terms of the mean and 95% CI per time, showing a steep increase in crystallinity up until 10 min, and then a saturation as the polymer layer is grown thicker and ordered to form HDPE-like PE, as seen in the spectral resemblance.

**Fig. 4:**
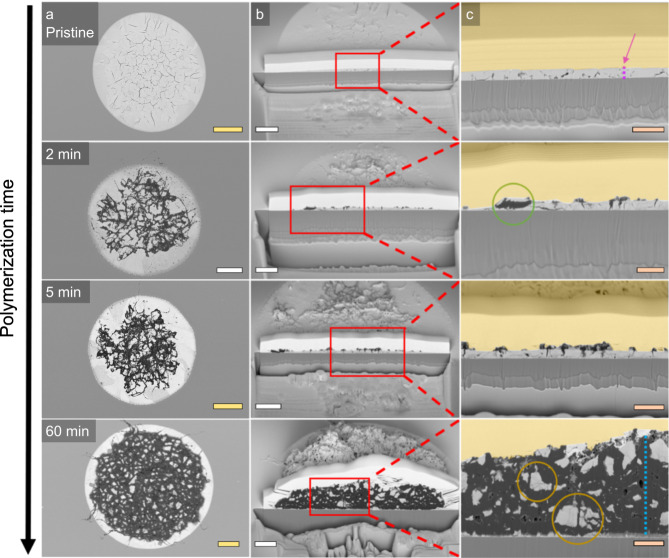
Cross-sectional scanning electron microscopy (SEM) to study the fragmentation behaviour of the ethylene polymerized LaOCl spherical caps. **a** Top-down overview of the full spherical caps at respectively 0 (pristine), 2, 5 and 60 min of ethylene polymerization. **b** A cross-section of the spherical caps that shows the internal structure of the polyethylene-LaOCl spherical cap composites. **c** A zoom-in of these cross-sections to provide enhanced view of the internal structure. The pink and blue dashed lines in respectively the pristine and 60 min ethylene polymerized samples show a thickness of 600 nm for the LaOCl spherical cap and 5.7 microns for the polyethylene layer at those positions. The green circle refers to LaOCl fragments having been lifted up from the spherical cap by the polyethylene formed within the porous catalyst framework. The orange circles indicate the internal cleavage sites of the LaOCl framework. The yellow scale bars represent 10 µm, the white scale bars 5 µm and the orange scale bars 2 µm. A yellow, transparent overlay is provided that indicates the coated Pt layer, all though in some cases it is observed to penetrate into the porous polyethylene layer.

In Fig. 2, an overview of the utilized micro-spectroscopic toolbox is given on a 20 min ethylene polymerized LaOCl spherical caps sample. Starting with ToF-SIMS, the composition of both the surface chemistry as well as the interior through depth profiling can be obtained. Here, a secondary electron image of an ethylene polymerized LaOCl spherical cap is shown together with the distribution of the negatively charged fragments of LaOCl^−^, TiOCl^−^ (due to exposure to moisture)_,_ and the polyethylene characteristic C_21_H_31_^−^. Additional ToF-SIMS results on a pristine LaOCl surface, different ethylene polymerization times and a HDPE reference film are given in the Supplementary Information section 3. With the vibrational micro-spectroscopy part of the toolbox, based on Raman microscopy and PiFM, the polyethylene phase is studied in-depth as shown in Figs. 2 and 3. Raman microscopy provides a time-efficient method to map the distribution of the -CH stretching vibrations in the region of 2700–3100 cm^−1^ to visualize local thickness differences of the formed polyethylene phase within the spherical caps. However, even in the best-case scenario, the diffraction-limited spatial resolution of the Raman microscope setup utilized here would be on the order of 360 nm (see Eq. 1). On the other hand, with PiFM, IR spectra with the spatial resolution of the AFM tip’s apex (∼20 nm) are obtained. This allows for the mapping and correlation of the -CH_2_- bending vibrations of crystalline polyethylene at respectively 1461 cm^−1^ (*B*_1u_) and 1471 cm^−1^ (*B*_2u_) to the topological information obtained from the AFM part^45^. Finally, FIB-SEM images provide strong *Z*-contrast between the low atomic weight polyethylene, the intermediate atomic weight Si(100) substrate and the high atomic weight LaOCl framework through the detection of backscattered electrons. Using both top-view and cross-sectional SEM images, the fragmentation of the LaOCl spherical cap as initiated by the induced stress of the polyethylene on the catalyst framework at respectively the spherical cap’s exterior surface and interior volume can be observed. An in-depth study on this fragmentation behaviour of the LaOCl spherical caps at different ethylene polymerization times will be given around Fig. 4.

### In-Depth Study on the Formation of Polyethylene

With the advanced micro-spectroscopy toolbox introduced for a 20 min ethylene polymerized LaOCl spherical cap, the formation of polyethylene will now be studied in-depth using the vibrational micro-spectroscopy part of this toolbox. To complement this 20 min ethylene polymerized sample, Raman microscopy and PiFM results of three additional ethylene polymerization times, namely 2, 5 and 60 min, are given in Fig. 3. The results on other polymerization times of respectively, 0 (pristine), 1 and 10 min are given in the Supplementary Information sections 2 and 4. In Fig. 3a, Raman microscopy results of the -CH stretch region at 2700–3100 cm^−1^ are given to map the distribution and thickness fluctuations of the formed polyethylene. Starting at the 2 min ethylene polymerization sample, the formation of polyethylene can clearly be observed both at the centre and edges of the spherical cap. Interestingly, at the edges of the spherical cap the Raman signal intensity characteristic for polyethylene is higher than at the centre. Upon increasing the polymerization time towards 5, 20 and finally 60 min we observe two trends. The first is that of a more complete, denser coverage of the formed polyethylene over the spherical cap and the second is the increase of the overall intensity of the polyethylene phase indicating the formation of thicker polyethylene layers and thus increased polyethylene yields as a function of polymerization time (increase in the contrast of darker background versus intense polyethylene signals as function of polymerization time). The Raman maps are nicely complemented by the high spatial resolution PiFM maps in Fig. 3b, which shows that already at the early polymerization times we can observe well-defined and intertwined polyethylene fibres growing outwards of the LaOCl spherical caps and extending towards the Si(100) substrate background. For the 60 min polymerized sample, at the bottom left and therefore close to the centre or thickest point of the spherical cap, a LaOCl fragment can be observed to lay partially within and on top of the polyethylene fibres. The polyethylene layer here reaches a thickness of up to 5 microns as shown in Fig. S9, which is pushing the limits of the experimental setup. Additional topological images of the full spherical caps and zoomed-in PiFM maps of all polymerization times are given in Supplementary Information section 4, showing the presence of polyethylene fibres at all polymerization times as well as the increase in the overall polyethylene thickness and thus polyethylene yield as a function of polymerization time.

In Fig. 3c, the IR spectra obtained from PiFM are given for all the polymerization times studied. The signal-to-noise-ratio for the 1 and 2 min ethylene polymerization time are quite low, which could be explained either by the formation of the highly porous and thin polyethylene fibres at such low polymerization times or that the crystallinity of the polyethylene fibres is lower at these earlier polymerization times, leading to a higher contribution of an amorphous polyethylene phase. As the polymerization time increases, the clear doublet peaks of crystalline polyethylene appear after roughly 5 min of polymerization.

Using multivariate curve resolution (MCR), the contribution of the -CH_2_- bending vibrations at 1461 cm^−1^ and 1471 cm^−1^ belonging to crystalline polyethylene in the orthorhombic phase and a broad amorphous band at 1463 cm^−1^ as well as an interference component due to adsorption of water vapour were assessed^46,47^. The scores of this MCR, which can be thought of as the concentration of a spectral component in the sum spectrum, were subsequently used to calculate the fraction of crystalline components to the overall intensity of the 1400–1500 cm^−1^ region (that is the two crystalline and one amorphous bands). This fraction as a function of ethylene polymerization time is shown in Fig. 3c. Initially, this fraction increases with polymerization time up to 10 min and then decreases and stabilizes for the 20 and 60 min samples. The increase of the fraction of crystalline bands as a function of polymerization time could be related to the polymerization rate where AFM and ToF-SIMS analysis in Figs. S8 and S11 show a decay in the increase of the estimated polyethylene on the external surface after roughly 2–5 min. This decrease of the polymerization rate, most likely due to internal mass transfer limitations within the LaOCl spherical caps, could then lead to a higher ratio of the rate of crystallization versus polymerization, since α-olefin polymerization is highly exothermic and can delay the crystallization of the formed polyolefin fibres. This would finally result in a higher fraction of crystalline components estimated with PiFM and subsequent MCR analysis with respect to the amorphous component. The decrease of the crystalline fractions for the 20 and 60 min polyethylene samples as compared to the 10 min polyethylene sample could be due to the increase of the chain length of the polyethylene phase as no chain-terminating reagent, such as H_2_, is employed. The increased polyethylene chain length leads to a decrease in the crystallization rate due to the increased viscosity of these longer polyolefin chains^48^. However, it should be emphasized here that no claims are made to the actual value of the crystallinity of the formed polyethylene phase, which is more conventionally assessed with bulk techniques such as differential scanning calorimetry requiring at least milligram quantities of polyethylene (DSC)^49^.

### On the fragmentation behaviour of the spherical cap catalyst

The vibrational microscopy part of the imaging toolbox confirmed the formation and evolution of the polyethylene crystallinity as a result of the ethylene polymerization reaction. Additionally, the formation of highly intertwined polyethylene fibres could be observed with the high spatial resolution PiFM maps, especially for the earliest polymerization times. To provide insights into the morphological evolution of the polyethylene phase as well as the fragmentation behaviour of the LaOCl support matrix, top-view and cross-sectional FIB-SEM is utilized. The SEM results of the 20 min ethylene polymerized LaOCl spherical cap sample are extended towards pristine, 2, 5, and 60 min in Fig. 4. Additional top-view and zoom-in SEM images of all ethylene polymerization times as well as EDS images are provided in the Supplementary Information section 2.

The observed fragmentation behaviour of these spherical caps consists of three stages as drawn schematically in Fig. S17. In the first stage, roughly 1 to 5 min of ethylene polymerization, the macroporous and surface-cracked LaOCl spherical cap comes in contact with ethylene that is transformed into polyethylene. Two different polymerization phenomena are observed in this first stage depending on the location within the spherical cap based on Figs. 4 and S19. Namely, (i) around the centre of the spherical cap, the first polyethylene fibres are extruded out of the surface exposed cracks and locally start to peel off the LaOCl framework, which becomes infused with the expanding polyethylene fibres. This peeling of small catalyst fragments belongs to the shrinking core fragmentation model and can clearly be observed in Fig. S19 for the zoom-ins on the 1 and 2 min polymerized samples (see Fig. S16). (ii) The formation of polyethylene within the internal macroporous cavities that leads to the rapid filling of such cavities. At the outer rim of the spherical caps this cavity filling is observed to lead to a significant degree of internal catalyst fragmentation already at this first stage. This type of catalyst fragmentation as described by the internal cleavage of the framework into successively smaller fragments belongs to the continuous bisection fragmentation model^50^. A visualization of the shrinking core and continuous bisection fragmentation models is given in Fig. S16. The second stage, roughly 10 to 20 min of ethylene polymerization, expands on the first phase through the steady growth of both formed polyethylene phenomena observed. That is, the porous polyethylene network formed due to the extrusion of polyethylene fibres from the exposed surface cracks increases both in total surface coverage as well as thickness and the thicker polyethylene regions observed at the outer rim, which cause a heavy local degree of framework fragmentation, increase further in thickness. Additionally, these thicker spheroidal regions, leading to a high degree of internal catalyst fragmentation, start to appear now also around the centre of the spherical cap as the build-up of stress exerted on the locally thicker LaOCl framework region has now surpassed the required threshold. The presence of these spheroidal regions that contain a thicker polyethylene phase were also observed earlier with the Raman microscopy maps in Figs. 3 and S5 as the regions with the higher signal intensity of the polyethylene characteristic -CH stretch vibrations. Finally, in the third stage, at 60 min of ethylene polymerization, the polymerization activity has been sufficiently high to cause a full disintegration of the original spherical cap morphology in both small and large LaOCl fragments, which however still show additional internal crack lines, dispersed uniformly throughout the formed polymer stage. The presence of these small and large LaOCl fragments with internal cleavage lines that are homogeneously dispersed throughout the formed polyethylene phase show that the continuous bisection fragmentation model has become the dominant fragmentation pathway for prolonged polymerization times.

Finally, to validate the LaOCl support matrix introduced in this study, a reference MgO/MgCl_2_ core-shell cap model system was prepared to support TiCl_4_. The MgO/MgCl_2_ core-shell approach was chosen such that the same procedure for the synthesis of spherical caps could be used as for LaOCl (e.g., conversion of a Mg^2+^ precursor to MgO under ambient conditions). Nonetheless, after chlorination of MgO towards a MgO/MgCl_2_ core-shell system, strong susceptibility towards moisture and hence morphology instability were observed with an AFM comparison when the same MgO/MgCl_2_ cap was measured under controlled (<1 ppm H_2_O) and ambient conditions in Fig. S14. Nonetheless, these reference spherical caps, which were grafted with TiCl_4_ and subsequently exposed to TEAL and ethylene under the same experimental conditions as for LaOCl showed a high ethylene polymerization activity. In Fig. S20, the SEM-EDX cross-sections are shown at different polymerization times. As expected, the MgO/MgCl_2_ exhibits a considerably weaker Z-contrast than LaOCl but in the cross-sections, especially for the 60 min polymerized sample the support matrix can still be identified within the polyethylene phase. The thickness of the 60 min polymerized MgO/MgCl_2_ support matrix is around 9 µm and therefore almost twice the thickness of that of the 60 min polymerized LaOCl support matrix indicating the considerably higher expected activity of MgCl_2_ supported TiC_4-x_ based on the similarities between Mg^2+^ and Ti^3+^/Ti^4+^ as mentioned earlier. The fragmentation behaviour of the MgO/MgCl_2_ clearly follows that of the continuous bisection model as observed already after 5 min of polymerization due to the high friability of the MgO/MgCl_2_ framework and even more obvious after 60 min of polymerization. However, it should be mentioned that due to the limited Z-contrast it is difficult to observe MgO/MgCl_2_ fragments that could be peeled off by polyethylene from the external surface.

In this work, a spherical cap model system has been introduced to bridge the gap between conventional planar model catalyst systems and the industrially used spherical catalyst particles in the field of α-olefin polymerization. Additionally, for this model system, LaOCl was chosen as a support matrix instead of the conventional MgCl_2_ support matrix to support the TiCl_4_ pre-active site. This provided both enhanced imaging contrast due to the high atomic weight of the lanthanum-based matrix and excellent moisture stability to enable the use of advanced micro-spectroscopy techniques under ambient conditions in contrast to the low atomic weight and highly hygroscopic MgCl_2_. X-ray photoelectron spectroscopy (XPS) gave evidence for the coordination of Ti^4+^ and even a minor presence of Ti^3+^ species on the LaOCl surface before contact with the triethylaluminium co-catalyst. Time-of-flight secondary ion mass spectrometry (ToF-SIMS) and Raman microscopy confirmed the LaOCl chemical phase on the spherical cap model as well as the formation of polyethylene at all polymerization times studied. Raman microscopy, photo-induced force microscopy (PiFM) and cross-sectional scanning electron microscopy (SEM) show the presence of two morphologically distinct polyethylene phases formed with (i) discrete and thicker polyethylene regions starting to be formed at the outer rim of the spherical cap at early polymerization times that gradually start to cover also the centre of the spherical caps and (ii) a network of highly intertwined polyethylene fibres distributed more uniformly over the spherical cap. Additionally, SEM shows that the polyethylene fibres are extruded out of crack lines already present on the pristine catalyst’s surface, which are subsequently expanding further due to the stress of the polymer induced on the framework. These extruded polyethylene fibres peel off small LaOCl fragments from the surface. At the outer rim of the spherical caps, large LaOCl fragments are lifted up from the framework due to the polymerization of ethylene within the LaOCl porous framework and confirms the observations made with Raman microscopy and PiFM of locally thicker and spheroidal polyethylene regions. These findings show that both limiting modes of fragmentation, namely the shrinking core (peeling off small fragments at the surface) and continuous bisection (internal cleavage of the framework) are occurring simultaneously throughout the polymerization process at early polymerization times. However, at 60 min ethylene polymerization, a full disintegration of the spherical cap morphology is observed according to a dominating continuous bisection fragmentation model. A reference MgO/MgCl_2_ cap system, which was observed to be highly susceptible to morphology changes upon exposure to moisture and possesses limited Z-contrast, showed a higher ethylene polymerization activity than the LaOCl framework but a similar dominant fragmentation pathway, namely that of continuous bisection. The use of this industrially relevant spherical cap model system can be expanded further for the high-throughput testing of support matrix formulations (both conventional and unconventional) in the field of α-olefin polymerization. These options include the use of highly tuneable and controllable deposition techniques such as inkjet printing as well as table-top SEM, Raman microscopy and scanning probe microscopy (AFM and PiFM) apparatus capable of fitting inside a glovebox or through the use of transfer vessels and inert measuring chambers^51^. Furthermore, semi-quantification of the Ti content through the use of additional highly sensitive imaging techniques such as µ-XRF or electron probe microanalysis could open the route to provide activity estimates of each individual spherical cap regarding ethylene polymerization. Finally, through modifying the polymerization reactor with high pressure and temperature capabilities as well as implementing mechanical overhead stirring to limit external mass transfer limitations combined with rapid quench methods, industrially relevant polymerization conditions could be mimicked^52,53^.

## Methods

### Material synthesis

B-doped Si(100) wafers with a thickness of 525 µm and resistivity of 0.005 Ohm_*_cm were purchased from Siegert Wafer. After cutting the wafers in suitable sizes (∼5 × 5 mm^2^ in size) with a diamond knife, a procedure for the grafting of the self-assembled monolayer and subsequent photo-patterning was followed as reported in literature^37^. First, the wafer pieces were cleaned with a 1:1:1 volumetric ratio of demineralized water, ethanol and acetone using an ultrasonicator for 15 min to remove surface contaminants. This step was then repeated in demineralized water for again 15 min. Then, the wafer pieces were placed in a beaker glass with a 5:1:1 volumetric ratio of demineralized water, ammonium hydroxide (28–32 wt% in H_2_O, Sigma-Aldrich) and hydrogen peroxide (30 wt%, Sigma-Aldrich) and placed on a hotplate set at 65 °C for 30 min. In this step, the native oxide surface layer on the Si(100) substrate is partially etched away and converted into surface hydroxyl groups. Afterwards, the wafers were first placed in a beaker of room-temperature demineralized water to quench this etching step and then heated to around 95 °C in demineralized water to remove any surface absorbed ammonium species for an additional 30 min. The wafer pieces are blown-dry with a N_2_ line and then placed in an oven at 120 °C for 1 h. This is followed by the transfer of the wafer pieces to a glovebox operating at <1 ppm O_2_ and H_2_O, where the wafer pieces were placed in a solution of 10 mM octadecyltrichlorosilane (99%, Sigma-Aldrich) in anhydrous toluene (99.9% stored over 4 Å molecular sieves, Sigma-Aldrich) for 10 min at room temperature. After the successful grafting of the self-assembled monolayer to the Si(100) surface hydroxyl groups, the wafer pieces were washed with anhydrous toluene and taken out of the glovebox. There they were blow-dried again with a N_2_ flow and placed at 120 °C for 5 min to anneal the ODTS layer. The wafer pieces were then placed in a UV/Ozone cleaner from Osilla Ltd with a custom-made 20 × 20 mm^2^ copper grid photomask with a 600 mesh size and circular holes from Agar scientific placed on top of them. A piece of 5 mm thick quartz glass was finally placed on top of the photomask and the UV/Ozone treatment is performed for 30 min.

Meanwhile, a 20 mM lanthanum chloride heptahydrate (99.9%, Sigma-Aldrich) solution in ethanol (99.9%, Sigma-Aldrich) was prepared for the spin-coating step. With the wafers patterned, they were transferred to a spin-coater from Ossila Ltd that uses a recessed spin-coating chuck to hold the wafers in place. Spin-coating was performed at 3000 rpm for 30 s with an injection volume of 10 µL on the wafer surface and a loading time of 5 s. After the spin-coating, the wafer pieces were transferred into a quartz boat that was subsequently placed inside a tube oven under dry N_2_ flow of 200 mL/min. The heat program consisted of three steps. First with a ramp of 2 °C/min to a temperature of 250 °C and dwell time of 4 h, followed by heating up to 600 °C with the same ramp and staying for an additional 4 h. Finally, the oven was cooled down 200 °C where it remained until the wafers were brought at this temperature into the glovebox. In the case of the MgO/MgCl_2_ reference sample, the same spin-coating and calcination recipe was used, including the direct transfer of the MgO intermediate chemical phase (from the calcination step at 200 °C) into the glovebox. For the spin-coating step, a 20 mM magnesium nitrate hexahydrate (99%, Sigma-Aldrich) solution in ethanol was prepared.

The grafting of the TiCl_4_ pre-active site onto the LaOCl spherical cap system has to be performed under stringent controls (<1 ppm O_2_/H_2_O) to prevent decomposition of TiCl_4_ and was done inside a glovebox. In a 20 mL glass vial, a 30 vol% solution of TiCl_4_ (99.9%, Sigma-Aldrich) in anhydrous heptane (99.9% stored over 4 Å molecular sieves, Sigma-Aldrich) was prepared to which the wafer pieces were added. The vial was then closed, wrapped in aluminium foil for better heat transfer, and put on a hotplate at 95 °C for 1 h. Afterwards, the vial was cooled down to room temperature naturally, after which the wafer pieces were transferred sequentially through three glass vials filled with pure heptane to remove excess TiCl_4_. In the last of the three washing vials, the wafers were kept for 30 min until they were transferred to a final fourth vial filled with heptane where they were kept for storage until commencing ethylene polymerization. In the case of the MgO/MgCl_2_ reference sample, the chlorination of the MgO spherical caps was performed based on the work of Chammingkwan et al.,^54^. However, instead of using pure TiCl_4_ under refluxing conditions, we chose to use the 30 vol% solution of TiCl_4_ in heptane at 95 °C for 1 h as performed also for the grafting of TiCl_4_ on LaOCl. Afterwards, the same washing steps were performed as for the LaOCl spherical cap samples and were kept in a vial filled with heptane for storage until commencing ethylene polymerization.

### Material testing

A custom-made low-pressure polymerization set-up was built to run inside the glovebox. This set-up allows us to either evacuate a cylindrical glass reactor with an internal volume of roughly 100 mL using an external vacuum pump or feed ethylene (4.5 N purity, Linde) at a pressure of 2 bar. The reactor was filled with a 10 mL anhydrous heptane solution containing 1 mg/mL triethylaluminium (98%, Sigma-Aldrich). The reactor was then slowly evacuated without stirring to remove the N_2_ atmosphere followed by feeding the ethylene at 2 bar until the solution became saturated. Meanwhile, the wafer pieces that now have the TiCl_4_ pre-active site grafted on the LaOCl surface were still stored in heptane. Two wafer pieces per polymerization time were then taken out and dried for 1 min under a N_2_ atmosphere. After saturating the solvent with ethylene, the reactor was slowly evacuated to atmospheric pressure, opened and quickly the two wafer pieces were placed inside the reactor followed by closing the reactor again. The reactor was then repressurized with ethylene in the absence of stirring and at this moment the timing of the polymerization reaction was commenced. The steps from placing the wafers in the ethylene saturated solution to closing the reactor and repressurizing typically took about 10 s. Quenching of the reaction was performed by quickly evacuating the reactor after the desired time, filling the reactor with N_2_, opening the reactor and placing the wafer pieces in a separate vial containing several mL of pure heptane. These vials were then taken out of the glovebox and placed inside an ultrasonicator for 90 s followed by drying under a mild N_2_ flow. The ultrasonicator step was found to be necessary to remove what would otherwise form a thick film originating from the hydrolysis of the co-catalyst after exposure to air. Afterwards, the polymerized wafers were dried overnight in a 60 °C oven and stored in a container labelled with the polymerization time.

### Focused ion beam-scanning electron microscopy

Focused ion beam scanning electron microscopy (FIB-SEM) images were collected on a FEI Helios NanoLab G3 UC scanning electron microscopy. The wafers of interest were placed on aluminium SEM stubs using a conductive carbon tape on the back-side of the wafer. For SEM images, the accelerating voltage was set to 2 kV and the current to 0.10 nA and the backscattered electron images were collected with a through-lens-detector to take full advantage of the strong *Z*-contrast between LaOCl, the formed polyethylene and the Si(100) background. EDX elemental mapping was performed with a silicon drift detector (SDD) X-MAX from Oxford Instruments. Prior to the milling of the region of interest with the focused ion beam a 3 µm thick Pt layer was deposited on top of the region of interest. The focus ion beam accelerating voltage was set at 30 kV and the current for both milling and cleaning at 0.43 nA. The milling was performed to create a trench perpendicularly to the surface and roughly at the centre of a spherical cap. After the milling step, the cross-section was cleaned with Ga ions before collecting the backscattered electron images.

### Photo-induced force microscopy

Photo-induced force microscopy (PiFM) micrographs, intensity maps and spectra were collected on a Molecular Vista Vistascope microscope equipped with a block engineering tuneable quantum cascade laser (QCL), having a 1965–785 cm^−1^ range. Gold coated tips (F = 100–130 N/m, resonance frequency >320 kHz) were used to obtain micrographs of the patterns of interest in semi-contact mode. Then, either the same ROI was scanned subsequently while changing the laser wavelength to 1471, 1461 and 1600 cm^−1^ to collect individual intensity maps, or the tip was brought to a position of choice and a full spectrum was recorded with 1 cm^−1^ resolution, 200 averages. The obtained height micrographs were post-processed in Gwyddion^55^. A plane background was subtracted over the Si(100)-substrate background, and the data were treated with a line-by-line correction using a “Trimmed mean of differences” function using a Trim faction of 0.5. The IR intensity maps were only processed by a line-by-line correction, and the maps were binned using a 2 pixel mean filter. To obtain a representative “average” PiF spectrum per PE time, 9 different patterns per time were partially mapped (∼10–20 lines) and 2 point spectra were recorded on PE features per pattern. In case of the MgO/MgCl_2_ reference sample, 10–15 spectra were recorded on 5 different patterns per time, and one of these representing patterns was measured with AFM as reference. The recorded point spectra per time (for both sample matrices) were pre-processed by applying a Whittaker baseline correction and normalization using the PLS Toolbox of Eigenvector. Then, the individual spectra were fitted with 4 components obtained from a MCR analysis (see Supplementary Information, Fig. S14). The resulting scores, representing the contribution of different components per spectra, were then used to determine the percentage of crystalline (MCR) component present in all individual spectra per polymerization time. Using the scores of these extracted spectral components of MCR, the fraction of the crystalline components (1461 cm^−1^ and 1471 cm^−1^) to that of the single amorphous component (1463 cm^−1^) was calculated as shown in Eq. 1.

1 $$	{{{{{\rm{Fraction}}}}}}\,{{{{{\rm{of}}}}}}\,{{{{{\rm{crystalline}}}}}}\,{{{{{\rm{components}}}}}}\, \\ 	=\frac{{Sum}\,{of}\,{crystalline}\,{polyethylene}\,{component}\,\left({{{{{\rm{\#}}}}}}1+{{{{{\rm{\#}}}}}}4\right)\,{scores}}{{Sum}\,{of}\,{all}\,{polyethylene}\,{component}\,\left({{{{{\rm{\#}}}}}}1+{{{{{\rm{\#}}}}}}3+{{{{{\rm{\#}}}}}}4\right)\,{scores}}\, .\,100\%$$

### Atomic force microscopy

Atomic force microscopy (AFM) was recorded on a Bruker MultiMode 8 with J Scanner in non-contact ScanAsyst HR mode, using silicon nitride ScanAsyst-HR tips (F = 0.4 N/m, frequency = 130 kHz). Per polymerization time, 6 different patterned catalysts were measured (Fig. S9). The data were post-treated as explained before for the PiFM maps. Furthermore, *x* and *y* cross-sections were then plotted through the middle-point of the catalyst caps, and the resulting profiles were fitted using a power function on the exposed uncovered catalyst cap (Fig. S10). Then, the height and cap-base diameter, or chord length, were measured to obtain the catalyst volume, which could be subtracted of the total measured volume (obtained using Gwyddions “Statistics” function) to yield the net polymer volume. More information can be found in the Supplementary Information. In the case of the MgO/MgCl_2_ reference sample, the initial scan was performed with an identical model (and experimental approach) installed inside a glovebox operating at <1 ppm O_2_ and H_2_O. The location of the pattern was determined with optical microscopy, and then the sample was transferred to the AFM outside the glovebox. The moment the sample left the glovebox was denoted as “0 min”. Further AFM scanning and post-treatment was done as described before.

### Raman microscopy

Raman spectra were collected with the XploRA^TM^ PLUS Raman Spectrometer – Confocal Raman Microscope from Horiba Scientific. At all times a 532 nm excitation laser and 100x objective with 0.9 NA was used together with a 1200 mm^−1^ grating and slit and hole sizes of respectively 200 µm and 500 µm. Knowing the NA of the objective and the wavelength of the laser, it is possible to calculate the diffraction limited lateral spatial resolution according to Eq. 2, which would be ∼360 nm for a λ = 532 nm and NA = 0.9^56^.

2 $${{{{{\rm{Spatial}}}}}}\,{{{{{\rm{resolution}}}}}}=\frac{0.61 \, {{{{{\rm{x}}}}}}\,{{\uplambda }}}{{NA}}$$

To collect high resolution maps, a laser power of 25% maximum (15.55 mW) was used with a 50 ms dwell time, 1 accumulation per spectrum, a step size of 0.25 µm and a scan range of 2700–3100 cm^−1^. To collect full-range, single point spectra, a laser power of 10% maximum (6.81 mW) was used with a 500 ms dwell time and 30 accumulations. Background corrections were performed within the Fityk software. No smoothing function was applied on the Raman spectra.

### Time-of-flight secondary ion mass spectrometry

Time-of-flight secondary ion mass spectrometry measurements (ToF-SIMS) were carried out with a M6 Hybrid SIMS (IONTOF GmbH, Münster, Germany). The machine is equipped with a 30 keV Bi nanoprobe primary ion gun, low energetic (2 kV) Cs and EI gas sputter guns as well as a 20 keV Arx/(O2)x gas cluster source. The samples were mounted with a double-sided copper tape on an electrical insulating glass slide.

Depth profiles were carried out with 30 keV Bi^3+^ primary ions in non-bunched mode (aperture 200 µm) in delayed extraction mode with topography option at negative polarity. The Bi^3+^ primary ion current was about 0.18 pA at 100 µs cycle time. The low energetic electron flood gun was used for charge compensation. Sputtering was done with a 10 keV Ar2000+ sputter beam (I ≃ 8 nA). Areas of 175 × 175 µm were probed in non-interlaced mode, with 512 × 512 pixels. The raster size of the sputter gun was 300 × 300 µm² centred to the analysis area. For data evaluation Surface Lab 7.2 (ION-TOF GmbH, Münster, Germany) was used. Mass calibration was carried out with the signals C^−^, OH^−^, C_2_^−^, Cl^−^, C_3_H_5_^−^, C_7_H_13_^−^, and LaOCl^−^. calibration and comparison to literature HDPE ToF-SIMS spectra was performed with respect to the work by Kern et al.^57^.

### X-ray photoelectron spectroscopy

X-ray photoelectron spectroscopy (XPS) measurements were performed on a PHI5000 Versa Probe II system from Physical Electronics. The X-ray source was monochromatic Al Kα radiation (1486.6 eV). The pass energy for detailed spectra in the analyzer was 23.50 eV. Data evaluation was performed with CasaXPS (version 2.3.22, Casa Software Ltd.). Energy calibration was performed based on the signal of the adventitious carbon at 284.8 eV. The following three bulk reference La powders were measured: La_2_O_3_ (99.9%, anhydrous powder, Sigma-Aldrich), LaCl_3_ (99.9+%, anhydrous powder, Sigma-Aldrich) and LaOCl (synthesized as described by Peringer et al.^58^). The XPS samples measured are based on the synthesis of LaOCl spherical caps with a 20 mM LaCl_3 *_7 H_2_O in ethanol solution as described earlier. These samples were measured as both pristine and after reaction with TiCl_4_ at 95 °C ex-situ. The in a N_2_-glovebox prepared samples were stored in heptane and transported in a sealed, steel vacuum tube to an Ar-filled glovebox. There they were opened, dried under Ar atmosphere and transferred into the XPS system using a transfer vessel under Ar atmosphere.

## Supplementary information

Supplementary Information

## Data Availability

The data that support the findings of this study are available from the corresponding author upon reasonable request.

## References

[1] Severn, J.R. and Chadwick, J.C. *Tailor-made Polymers: Via Immobilization of Alpha-Olefin Polymerization Catalysts Ch. 2* (Wiley-VCH, Weinheim, 2008).

[2] Kaminsky, W. *Polyolefins: 50 years after Ziegler and Natta I Ch. 2* (Springer, Berlin, Heidelberg, 2013).

[3] Albunia, A.R., Prades, F., Jeremic, D. *Multimodal Polymers with Supported Catalysts Ch. 1* (Springer Nature Switzerland, 2019).

[4] Hoff, R. *Handbook of Transition Metal Polymerization Catalysts Ch. 6*, (John Wiley & Sons, Hoboken, 2018).

[5] Piovano A, Zarupski J, Groppo E (2020). Disclosing the Interaction between Carbon Monoxide and Alkylated Ti^3+^ Species: a Direct Insight into Ziegler-Natta Catalysis. J. Phys. Chem. Lett..

[6] Busico V (1999). High-resolution 13C NMR configurational analysis of polypropylene made with MgCl_2_-supported Ziegler-Natta Catalysts. 1. The “Model” System MgCl_2_/TiCl_4_−2,6-Dimethylpyridine/Al(C_2_H_5_)_3_. Macromol.

[7] Brambilla L, Zerbi G, Piemontesi F, Nascetti S, Morini G (2007). Structure of MgCl_2_-TiCl_4_ complex in co-milled Ziegler-Natta catalyst precursors with different TiCl_4_ content: experimental and theoretical vibrational spectra. J. Mol. Catal. A Chem..

[8] Vittoria A, Meppelder A, Friederichs N, Busico V, Cipullo R (2017). Demystifying Ziegler-Natta catalysts: the origin of stereoselectivity. ACS Catal..

[9] Piovano A (2021). Electronic properties of Ti sites in Ziegler-Natta Catalysts. ACS Catal..

[10] Sauer J, Freund HJ (2015). Models in catalysis. Catal. Lett..

[11] Gao F, Goodman DW (2012). Model catalysts: simulating the complexities of heterogeneous catalysts. Annu. Rev. Phys. Chem..

[12] Kim HS, Tewell CR, Somorjai GA (2002). Surface science studies of Ziegler-Natta olefin polymerization system: correlations between polymerization kinetics, polymer structures, and active site structures on model catalysts. Korean J. Chem. Eng..

[13] Magni E, Somorjai GA (1995). Preparation of a model Ziegler-Natta catalyst Surface science studies of magnesium chloride thin film deposited on gold and its interaction with titanium chloride. Appl. Surf. Sci..

[14] Magni E, Somorjai GA (1996). Preparation of a model Ziegler-Natta catalyst: electron irradiation induced titanium chloride deposition on magnesium chloride thin films grown on gold. Surf. Sci..

[15] Magni E, Somorjai GA (1996). Electron irradiation induced chemical vapor deposition of titanium chloride on gold and on magnesium chloride thin films. Surface characterization by AES, XPS, and TPD. J. Phys. Chem. B.

[16] Magni E, Somorjai GA (1997). Surface science study of model Ziegler-Natta catalysts. Surf. Sci..

[17] Arlman EJ, Cossee P (1964). Ziegler-Natta catalysis III. Stereospecific polymerization of propene with the catalyst system TiCl_3_lEt_3_. J. Catal..

[18] Siokou A, Ntais S (2003). Towards the preparation of realistic model Ziegler-Natta catalysts: XPS study of the MgCl_2_/TiCl_4_ interaction with flat SiO_2_/Si(100). Surf. Sci..

[19] Siokou A, Ntais S (2006). XPS investigation of the interaction between TiCl_4_(THF)_2_ and AlEt_3_ modified SiO_x_/Si(100) surfaces. Surf. Sci..

[20] Giannini, U., Albizatti, E., Parodi, S., Pirinoli, F., Catalysts for polymerizing olefins, U.S. Patent 4,124,532 (1978).

[21] Albunia, A.R., Prades, F., Jeremic, D. *Multimodal Polymers with Supported Catalysts Ch. 2* (Springer Nature Switzerland, 2019).

[22] Bart JCJ, Roovers W (1995). Magnesium chloride – ethanol adducts. J. Mat. Sci..

[23] Andoni A, Chadwick JC, Milani S, Niemantsverdriet HJW, Thüne PC (2007). Introducing a new surface science model for Ziegler-Natta catalysts: Preparation, basic characterization and testing. J. Catal..

[24] Andoni A, Chadwick JC, Niemantsverdriet HJW, Thüne PC (2007). A preparation method for well-defined crystallites of MgCl_2_-supported Ziegler-Natta catalysts and their observation by AFM and SEM. Macromol. Rap. Comm..

[25] Andoni A, Chadwick JC, Niemantsverdriet HJW, Thüne PC (2008). The role of electron donors on lateral surfaces of MgCl_2_-supported Ziegler-Natta catalysts: Observation by AFM and SEM. J. Catal..

[26] Andoni A, Chadwick JC, Niemantsverdriet HJW, Thüne PC (2009). Investigation of planar Ziegler-Natta model catalysts using attenuated total reflection infrared spectroscopy. Catal. Lett..

[27] Piovano A, Thushara KS, Morra E, Chiesa M, Groppo E (2016). Unraveling the catalytic synergy between Ti^3+^ and Al^3+^ sites on a chlorinated Al_2_O_3_: a tandem approach to branched polyethylene. Angew. Chem..

[28] Piovano A, Morra E, Chiessa M, Groppo E (2017). Tuning the Ti^3+^ and Al^3+^ synergy in a Al_2_O_3_/TiCl_x_ catalyst to modulate the grade of the produced polyethylene. ACS Catal..

[29] Jones, M.E., Olken, M.M., Hickman, D.A. Process for vinyl chloride manufacture from ethane and ethylene with secondary reactive consumption of reactor effluent HCl, WO Patent 2001038273 A1 (2001).

[30] Schweizer, A.E., Jones, M.E., Hickman, D.A. Oxidative halogenation of C1 hydrocarbons to halogenated C1 hydrocarbons and integrated processes related thereto, U.S. Patent 6,452,058 B1 (2002).

[31] Van der Avert P, Weckhuysen BM (2002). Low-temperature destruction of chlorinated hydrocarbons over lanthanide oxide based catalysts. Angew. Chem. Int. Ed..

[32] Manoilova OV (2004). Surface acidity and basicity of La_2_O_3_, LaOCl and LaCl_3_ characterized by IR spectroscopy, TPD, and DFT calculations. J. Phys. Chem. B.

[33] Van der H (2005). Destructive adsorption of CCl_4_ over lanthanum-based solids: linking activity to acid-base properties. J. Phys. Chem. B.

[34] Podkolzin SG, Manoilova OV, Weckhuysen BM (2005). Relative activity of La_2_O_3_, LaOCl, and LaCl_3_ in reaction with CCl_4_ studies with infrared spectroscopy and density functional theory calculations. J. Phys. Chem. B.

[35] Podkolzin SG, Stangland EE, Jones ME, Peringer E, Lercher JA (2007). Methyl chloride production from methane over lanthanum-based catalysts. J. Am. Chem. Soc..

[36] Nowak D (2016). Nanoscale chemical imaging by photoinduced force microscopy. Sci. Adv..

[37] Masuda Y, Sugiyama T, Lin H, Seo WS, Koumoto K (2001). Selective deposition and micropatterning of titanium dioxide thin film on self-assembled monolayers. Thin Solid Films.

[38] Ha K (2001). Photochemical pattern transfer and patterning of continuous zeolite films on glass by direct dipping in synthesis gel. Adv. Mater..

[39] Van Delft FCMJM, van den Heuvel FC, Kuiper AET, Thüne PC, Niemantsverdriet JW (2004). Micro-contact printing on oxide surface for model catalysts using e-beam written masters in hydrogen silsesquioxane. Microelectron. Eng..

[40] Hölsä J, Lathinen M, Lastusaari M, Valkonen J, Viljanen J (2002). Stability of rarte-earth oxychloride phases: bond valence study. J. Solid State Chem..

[41] Renero-Lecuna C (2021). Nd^3+^-doped lanthanum oxychloride nanocrystals as nanothermometers. J. Phys. Chem. C..

[42] Zhu X (2017). Synthesis, characterization and mechanism of formation of carbon aerogels incorporated with highly crystalline lanthanum oxychloride particles. RSC Adv..

[43] Alizadeh S, Mousavi-Kamazani M, Salavati-Niasari M (2015). Hydrothermal synthesis of rod-like LaOCl nanoparticles from new precursors. J. Clust. Sci..

[44] Giunchi G, Allegra J (1984). Structural disorder in microcrystalline MgCl_2_. J. Appl. Cryst..

[45] Raff, R.A.V., Doak, K.W. *Crystalline Olefin Polymers Part 1*, (John Wiley and Sons Inc., New York, 1965).

[46] Hagemann H, Snyder RG, Peacock AJ, Mandelkern L (1989). Quantitative infrared methods for the measurement of crystallinity and its temperature dependence: polyethylene. Macromol.

[47] De Juan A, Jaumot J, Tauler R (2014). Multivariate Curve Resolution (MCR). Solving the mixture analysis problem. Anal. Methods.

[48] Whiteley, K.S., *Ch1*, In: *Ullmann’s Encyclopedia of Industrial Chemistry* (Wiley-VCH, Weinheim, 2005).

[49] Li D, Zhou L, Wang X, He L, Yang X (2019). Effect of crystallinity of polyethylene with different densities on breakdown strength and conductance property. Materials.

[50] Horácková B, Grof Z, Kosek J (2007). Dynamics of fragmentation of catalyst carries in catalytic polymerization of olefins. Chem. Eng. Sci..

[51] Liu X (2012). Inkjet printing assisted synthesis of multicomponent mesoporous metal oxides for ultrafast catalyst exploration. Nano Lett..

[52] Liu B, Matsuoka H, Terano M (2001). Stopped-flow techniques in Ziegler-catalysis. Macromol. Rapid Comm..

[53] Di Martino A (2005). Quenched-flow device for the characterisation of the nascent polymerisation of ethylene under industrial conditions. Macromol. Rapid Comm..

[54] Chammingkwan P, Than VQ, Terano M, Taniike T (2014). MgO/MgCl_2_/TiCl_4_ core-shell catalyst for establishing structure-performance relationship in Ziegler-Natta olefin polymerization. Top. Catal..

[55] Nečas D, Klapetek P (2012). Gwyddion: an open-source software for SPM data analysis. Cent. Eur. J. Phys..

[56] Graefe CT (2019). Far-field super-resolution vibrational spectroscopy. Anal. Chem..

[57] Kern S, Kern C, Rohnke M (2019). Mass spectra database of polymers for bismuth-cluster ToF-SIMS. Surf. Sci. Spectra.

[58] Peringer E, Tejuja C, Salzinger M, Lemonidou AA, Lercher JA (2008). On the synthesis of LaCl_3_ catalysts for oxidative chlorination of methane. Appl. Catal. Gen..

