# Supplementary material for: A Ziegler-type spherical cap model reveals early stage ethylene polymerization growth versus catalyst fragmentation relationships
Source: Nat Commun. 2022 Aug 24;13:4954. doi: 10.1038/s41467-022-32635-z (PMC9402950; doi:10.1038/s41467-022-32635-z)
Supplement: Supplementary file 1 — Supplementary Information [file 41467_2022_32635_MOESM1_ESM.pdf]

Supplementary Information

A Ziegler-type Spherical Cap Model Reveals Early Stage Ethylene Polymerization Growth Versus Catalyst Fragmentation Relationships

Koen W. Bossers[1,δ], Laurens D.B. Mandemaker[1,δ], Nikolaos Nikolopoulos[1], Yuanshuai Liu[1,2], Marcus Rohnke[3,4], Peter de Peinder[5], Bas J.P. Terlingen[1], Felix Walther[3,4], Joren M. Dorresteijn[1], Thomas Hartman[1], Bert M. Weckhuysen[*,1]

[1] Inorganic Chemistry & Catalysis, Debye Institute for Nanomaterials Science, Utrecht University, Universiteitsweg 99, 3584 CG Utrecht, The Netherlands

[2] Qingdao Institute of Bioenergy and Bioprocess Technology, Chinese Academy of Sciences, Songling Road 189, 266101 Qingdao, China

[3] Center for Materials Research, Justus Liebig University, Heinrich-Buff-Ring 16, 35392 Giessen, Germany

[4] Institute of Physical Chemistry, Justus Liebig University, Heinrich-Buff-Ring 17, 35392 Giessen, Germany

[5] VibSpec, Haaftenlaan 28, 4006 XL Tiel, The Netherlands

δ These authors contributed equally to this work

[*]email: b.m.weckhuysen@uu.nl

1. X-ray Photo-electron Spectroscopy

The La 3$d_{5/2}$, La 4*p* and Cl 2*p* X-ray photo-electron spectroscopy (XPS) data of the three reference materials, namely La_2O_3, LaOCl and $LaCl_3$, are given in Figure S1. Li *et al.,* have shown in an *in-situ* study on the energy calibration of Lanthanum compounds with XPS that the component splitting (ΔE) of the La 3$d_{5/2}$ signal, which in principle can be used for chemical diagnostics, is highly prone to the measurement conditions and surface chemistry [1]. They found, for instance, that the ΔE value of an as-prepared La_2O_3 material changed from 3.6 eV to 4.3 eV after vacuum treatment at 800 °C, which basically removes carbon and oxygen adsorbates. Furthermore, they provide a literature summary of reported ΔE values for La_2O_3, $La(OH)_3$ and $La_2O_2(CO)_3$ where differences up to 0.7 eV are reported within the same materials. Therefore, although the ΔE values of the three reference materials are provided in Figure S1 and a trend is observed showing a decrease in the splitting value with increasing degree of surface chlorination from 4.05 eV for La_2O_3 to 3.99 eV for LaOCl and finally 3.72 eV for $LaCl_3$ the absolute values should be taken with caution. Additionally, the use of the La 4*p* and Cl 2*p* XPS signals to follow the surface chemistry of the LaOCl spherical caps before and after reaction with $TiCl_4$ is highly challenging due to massive signal interference (strong overlap) of the La 4*p* and the Cl 2*p* XP signals. This prevents the determination of an accurate fitting model for the LaOCl spherical cap samples, since the individual signal features of the La 4*p* signal (e.g., the position of the high binding energy shoulder), which depend on the exact chemical environment (e.g., the degree of surface chlorination), cannot be properly determined. However, to provide rough trends on the degree of surface chlorination, Table S1 summarizes the results of a simplified fitting approach, neglecting the La 4*p* signal shoulder at high binding energies. Assuming that this leads to a systematic error (a systematic underestimation of the La 4*p* signal contribution), it should still be possible to derive trends related to the Cl fraction. Again, it should be noted here that the absolute values should be considered with caution due to the aforementioned fitting issues.

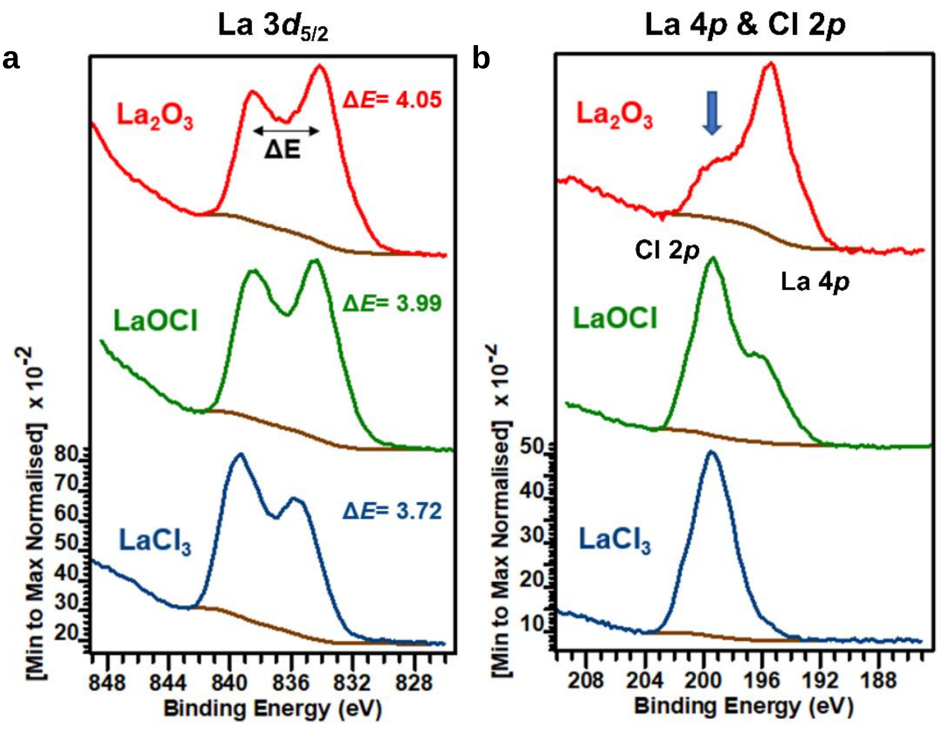

Figure S1. X-ray photo-electron spectroscopy (XPS) results of three Lanthanum reference materials. (a) The multiplet split of the La 3$d_{5/2}$ signals are shown for the anhydrous reference materials (La_2O_3, LaOCl and $LaCl_3$) together with the split energy, ΔE, in electron volts. (b) The La 4*p* and Cl 2*p* signals of the three reference materials. The blue arrow indicates the La 4*p* signal shoulder at high binding energies for La_2O_3 that overlaps with the Cl 2*p* signals for the chlorinated samples.

The La 3$d_{5/2}$, La 4*p* and Cl 2*p* XPS results on the LaOCl spherical caps before (SC-P) and after (SC-Ti) treatment with $TiCl_4$ at 95 °C are given in Figure S2. As discussed earlier, the ΔE splitting value is highly sensitive to the local chemical environment and the sample preparation steps. Since Δ*E* is approximately the same before and after the reaction of the LaOCl spherical cap with $TiCl_4$ (4.32 eV vs. 4.30 eV respectively) and significantly lower than the multiplet split difference between the LaOCl and the $LaCl_3$ reference powders (Δ(Δ*E*) = 0.27 eV) and therefore the LaOCl surface of the spherical cap doesn't seem to be chlorinated further towards $LaCl_3$ upon treatment with $TiCl_4$ at 95 °C.

The areas of the XPS signal contributions assigned to Cl, La and Ti of the spherical cap samples are summarized in Table S1. As expected, the Ti 2*p* XP signal is only observed after the reaction of the LaOCl spherical caps with $TiCl_4$. The broadening of the Ti 2$p_{3/2}$ signal towards lower binding energies (~456-457 eV) hints clearly towards the presence of reduced Ti^{3+} species [2]. This could be due to a similar mechanism reported for the dissociative chemisorption of CCl_4 on the LaOCl surface, which

gives rise to a $CCl^{\delta+}$-$Cl^{\delta-}$ pair coordinated to respectively the Lewis acid La^{3+} and Lewis basic O^{2-} surface groups [3,4]. However, it should be mentioned here that the majority of the Ti species remains in the +4 oxidation state in the absence of the co-catalyst, triethylaluminium. To exclude a dominating detrimental influence by the uncovered silicon areas on the LaOCl spherical cap sample in this context, a Si(100) substrate reference was treated with both $TiCl_4$ and the reducing triethylaluminium co-catalyst and subsequently analyzed with XPS. The corresponding Ti 2*p* signal is shown in blue in Figure S2c (denoted as Si-Ti/Al). For the Si-Ti/Al reference, the presence of reduced Ti species can also be observed, which can be attributed to the interaction with the reducing co-catalyst. However, the fraction of the reduced signal contributions is 3.4 times for the LaOCl SC-Ti sample than for the Si-Ti/Al reference. Therefore, the reduced Ti species can be mainly attributed to interactions with the LaOCl spherical caps.

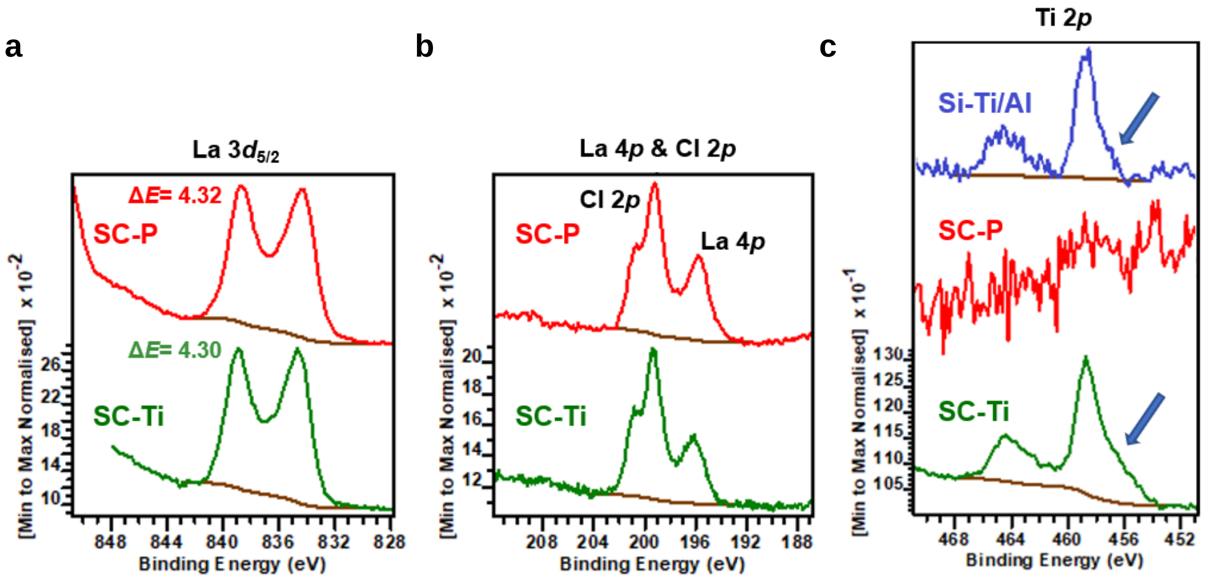

Figure S2. X-ray photo-electron spectroscopy (XPS) results of the LaOCl spherical cap model system before and after reaction with $TiCl_4$. (a) The multiplet split of the La $3d_{5/2}$ spectrum is given for the LaOCl spherical cap model system before (SC-P, red) and after (SC-Ti, green) reaction with $TiCl_4$. (b) The La 4*p* and Cl 2*p* spectra of SC-P and SC-Ti. (c) The Ti 2*p* spectrum of SC-P, SC-Ti and Si-Ti/Al (blue). The blue arrows show the presence of reduced Ti^{x+} species.

The ratios of Cl:La-related signals (Cl 2*p*:La $3d_{5/2}$ and Cl 2*p*:La 4*p*) of the pristine SC-P sample are slightly higher than those of the LaOCl reference, but considerably lower than those of the pure $LaCl_3$ reference. The slight deviation from the LaOCl reference might be caused by topography-related

effects and the different sample geometry (spherical caps versus powder references). However, after reaction with $TiCl_4$ the Cl:La ratios increase significantly. Here, due to massive signal interference, it is not entirely clear, whether this is due to a further chlorination of LaOCl towards $LaCl_3$ with $TiCl_4$ acting as the chlorination reagent or simply due to the chemisorption of $TiCl_x$ species on the external surface of LaOCl. Since the La $3d_{5/2}$ splitting value is not affected by the reaction with $TiCl_4$ as mentioned earlier, it seems more likely that the increased ratio of the Cl:La peaks is due to the chemisorption of $TiCl_x$ species.

Table S1. The area under the selected La, Cl and Ti XP signals and the respective signal ratios. Values are given for the three reference materials La_2O_3, LaOCl and $LaCl_3$ as well as the pristine and $TiCl_4$ treated LaOCl spherical cap systems called SC-P and SC-Ti, respectively.

Sample	Area La $3d_{5/2}$	Area La 4*p**	Area Cl 2*p*	Area Ti 2*p*	Ratio Cl 2*p* : La $3d_{5/2}$	Ratio Cl 2*p* : La 4*p*	Ratio Cl 2*p* : Ti 2*p*
**La_2O_3**	232.3	364.2	-	-	-	-	-
**LaOCl**	269.2	406.1	337.5	-	1.3	0.8	-
**$LaCl_3$**	168.3	185.0	525.1	-	3.1	2.8	-
**SC-P**	65.7	103.6	104.4	0.0	1.6	1.0	-
**SC-Ti**	38.2	61.2	79.9	81.8	2.1	1.3	1.0

* The area under the La 4*p* is obtained using a single signal (GL(30) line shape) and doesn't take the high binding energy shoulder observed around 200 eV into account.

2. Raman Micro-Spectroscopy

In Figure S3, the Raman spectra are given for the pristine LaOCl spherical cap, the Si (100) wafer background and the LaOCl powder reference material also used as the XPS reference. For the spherical cap model system, only the LaOCl chemical phase is detected. Conventional Raman micro-spectroscopy (e.g., in the absence of surface-enhancement techniques) is a bulk-probing technique that nicely complements the surface-sensitivity of XPS analysis in this scenario to study the spherical cap model system.

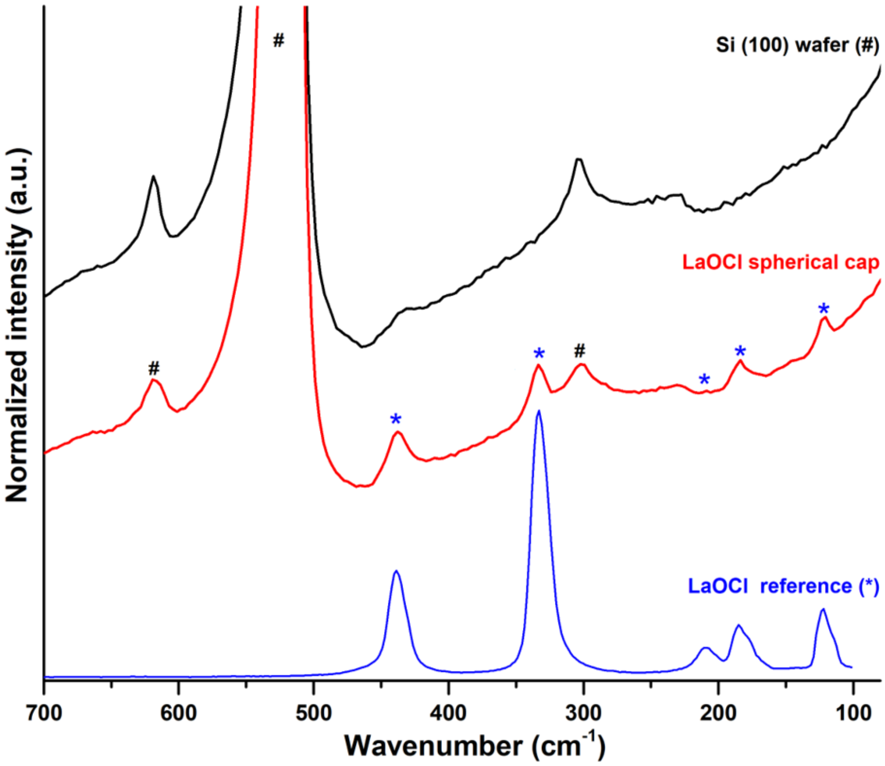

Figure S3. Raman micro-spectroscopy to determine the bulk chemical phase of the LaOCl spherical cap model system. In blue and black, the Raman spectra are given for respectively the LaOCl powder reference that was also used for XPS studies and a clean Si(100) wafer substrate. In red, the Raman spectrum is given for the LaOCl spherical cap model system, where the stars denote peaks belonging to the LaOCl chemical phase based on the blue spectrum and the octothorp denotes peaks belonging to the Si(100) wafer substrate.

In Figure S4, the Raman spectra are given for the LaOCl spherical caps after specified (0,1,2,5,10,20 and 60) min of ethylene polymerization, as well as the Si (100) wafer background on the 20 min ethylene polymerized sample, a pristine LaOCl spherical cap (without $TiCl_4$) but with TEAL after 60 min of ethylene polymerization and a HDPE reference film. All Raman spectra are normalized to the 521 cm^{-1} peak of the Si (100) substrate, which can be regarded as an internal standard. For all samples, the measured spot was optimized based on that part of the spherical cap that gave the most intense polyethylene signal. For all LaOCl/$TiCl_4$/TEAL ethylene polymerized samples, Raman micro-spectroscopy confirms that polyethylene is formed mainly on the LaOCl spherical caps, with no observable amount detected on the background substrate and no polyethylene or other carbon phases are found on the pristine sample (0 min PE). The LaOCl/TEAL sample without $TiCl_4$ treatment and after 60 min of ethylene polymerization showed no formation of polyethylene.

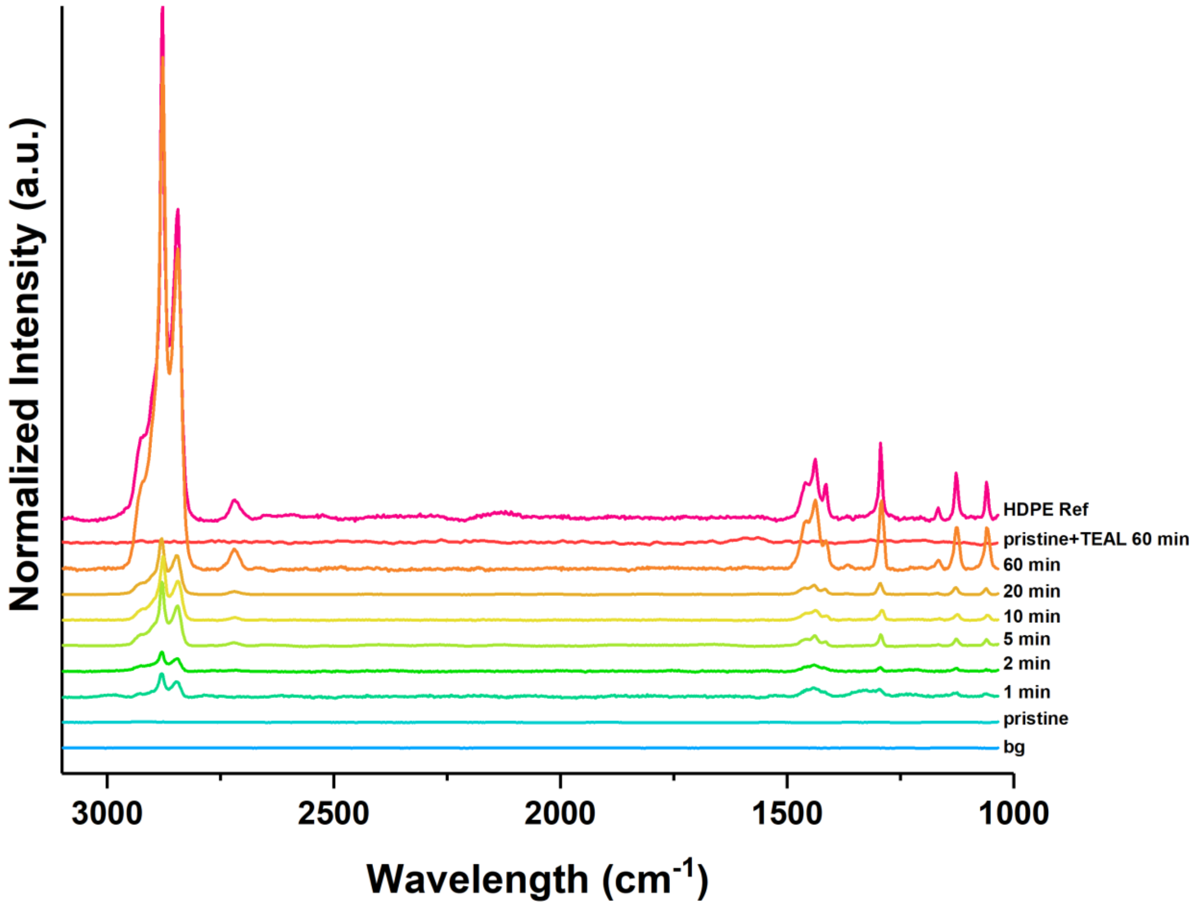

Figure S4. Raman spectra taken after different ethylene polymerization times on the LaOCl spherical caps as well as a HDPE reference film. Raman spectra are given from the pristine up to 60 min of ethylene polymerization on the LaOCl spherical cap model system (labelled as *x min PE*) as well as the Si (100) background of the 20 min ethylene polymerized sample (background 20 min PE), a pristine LaOCl spherical cap (without $TiCl_4$) but with TEAL after 60 min of ethylene polymerization and a HDPE reference film. In all cases, normalization was performed using the Si (100) background 521 cm^{-1} peak as an internal standard.

The strength of Raman micro-spectroscopy is the ability to differentiate and map chemical phases over the region of interest. In this case, it allows us to visualize the distribution of polyethylene on the LaOCl spherical caps at different polymerization times. In Figure S5, the Raman micro-spectroscopy maps of the 2700-3100 cm^{-1} are given, which visualize the asymmetric and symmetric $-CH_2-$ stretching modes of polyethylene. Unlike the normalized spectra in Figure S4, using the Si(100) 512 cm^{-1} substrate peak, these maps aren't normalized since this internal standard vibration fell outside of the imaged region. The inset in each Raman micro-spectroscopy map is the correlated optical microscopy image that shows the contours of the LaOCl spherical caps imaged. As shown in Figure S5, the pristine sample shows no detectable formation of polyethylene or other carbon phase with $-CH_2-$

groups that would fall in the same imaged region. Starting at the 1 min ethylene polymerized sample, we mainly observe polyethylene fibres distributed on the centre of the spherical cap with a few hotspots at the edge. The next samples, going from 2 min to 20 min ethylene polymerization, continue the trend with the formation of a polyethylene fibre network in the centre of the spherical caps and the formation of dense polyethylene regions around the edges. For both the 10 and 20 min ethylene polymerization samples, dense regions also start to appear in the centre of the spherical caps. Finally, the 60 min ethylene polymerization sample still shows some polyethylene fibres on top of the spherical cap but the dense regions have grown significantly enough in size to almost fully cover the spherical cap. For the 5, 20 and 60 min ethylene polymerized imaged spherical caps long polyethylene fibres spanning over 10 microns in length can be observed to grow away from the spherical cap and lay on the Si (100) substrate background.

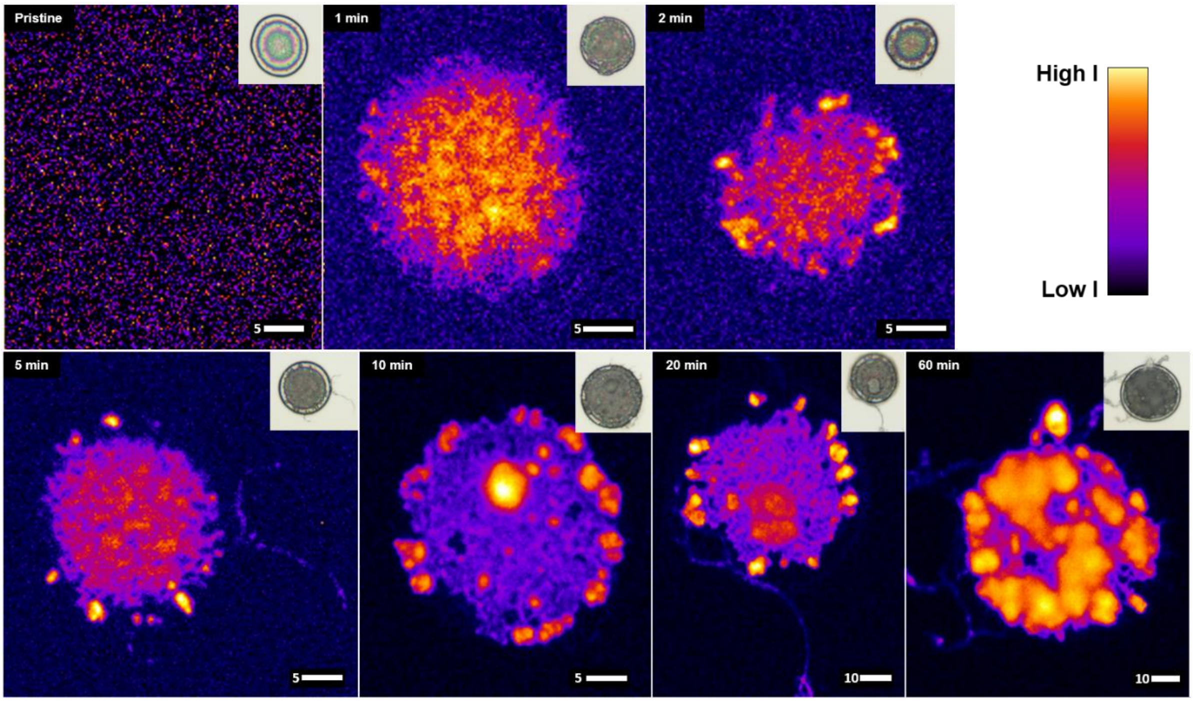

Figure S5. Raman micro-spectroscopy maps of the 2700-3100 cm^{-1} region on different ethylene polymerization times. The 2700-3100 cm^{-1} region imaged for each ethylene polymerization time shows the distribution of the asymmetric and symmetric $-CH_2-$ stretching modes of polyethylene on a LaOCl spherical cap. The inset images show the correlated optical microscopy overview of the pristine and ethylene polymerized LaOCl spherical caps. The scale bars depict the size in microns.

In Figure S6 the Raman spectra are given for the pristine and ethylene polymerized reference $MgO/MgCl_2$ caps. Starting with the 2 min ethylene polymerized sample, the polyethylene characteristic peaks, especially around 2700-2900 cm^{-1} are clearly visible.

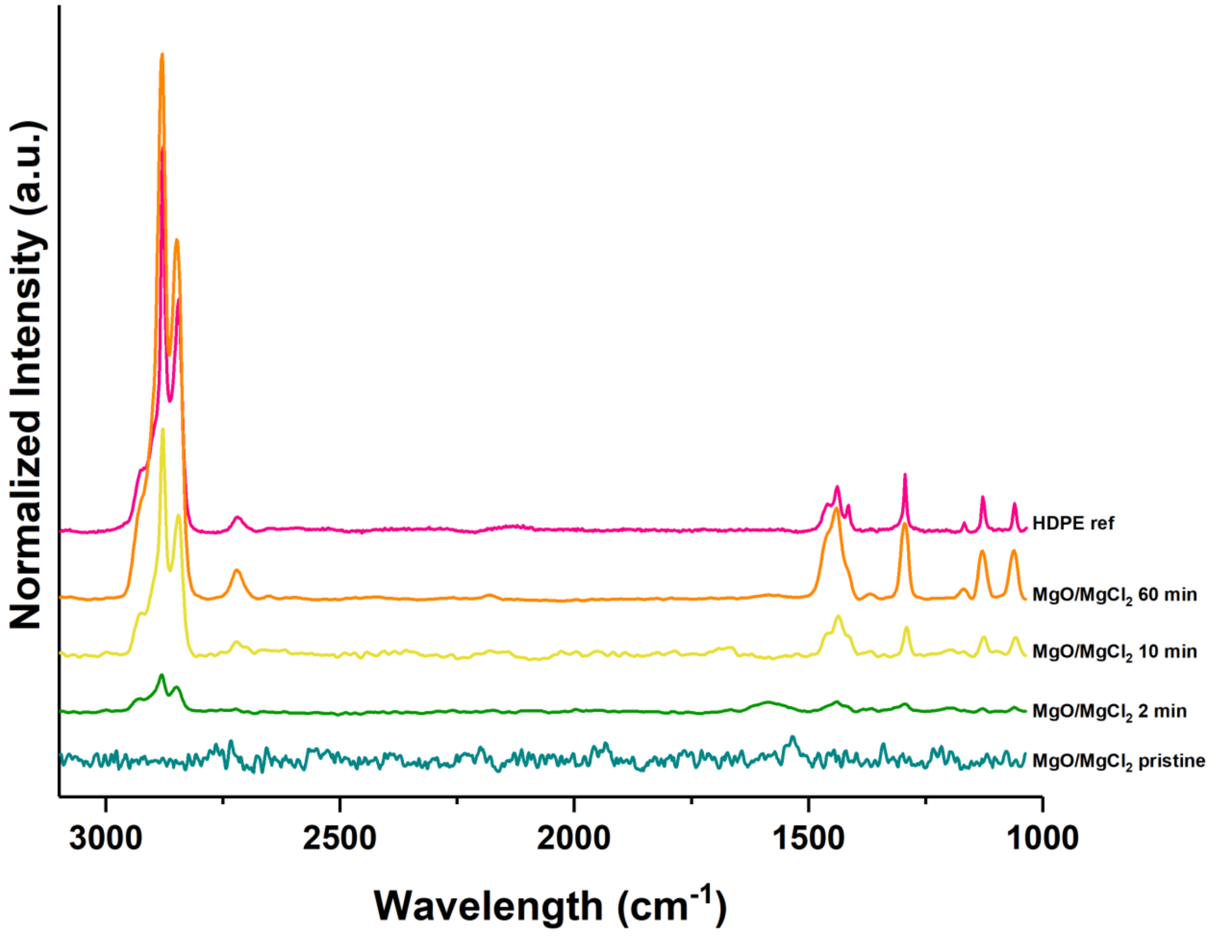

Figure S6. Raman spectra taken after different ethylene polymerization times on the reference $MgO/MgCl_2$ caps as well as a HDPE reference film. Raman spectra are given from the pristine up to 60 min of ethylene polymerization on the reference $MgO/MgCl_2$ cap samples (labelled as *$MgO/MgCl_2$ X min*) and a HDPE reference film. In all cases, normalization was performed using the Si (100) background 521 cm^{-1} peak as an internal standard.

In Figure S7 the Raman maps are given of the 2, 10 and 60 min ethylene polymerized reference $MgO/MgCl_2$ caps. The 2700-3100 cm^{-1} region is portrayed that visualizes the asymmetric and symmetric -CH_2- stretching modes of polyethylene. Whereas only a weak signal for polyethylene is observed for the 2 min polymerized sample, which is in agreement with the spectrum given in Figure S6, strong signals are observed for both the 10 and 60 min polymerized samples. Furthermore, whereas for LaOCl

spherical caps both small yet intense spheroidal regions are observed and a fibre-like weaker intensity region, for the $MgO/MgCl_2$ samples a more homogeneous polyethylene phase is observed of strong intensity.

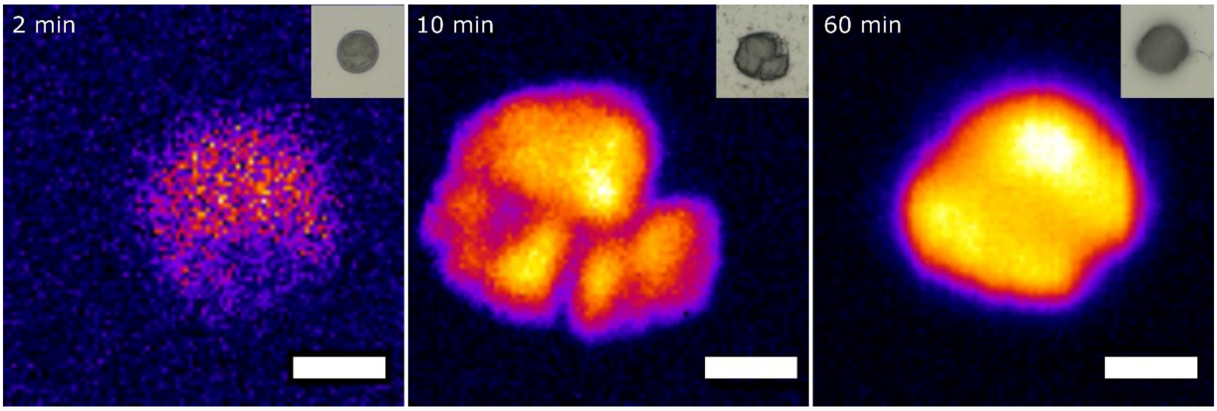

Figure S7. Raman micro-spectroscopy maps of the 2700-3100 cm^{-1} region on different ethylene polymerization times of the reference $MgO/MgCl_2$ caps. The 2700-3100 cm^{-1} region imaged for each ethylene polymerization time shows the distribution of the asymmetric and symmetric $-CH_2-$ stretching modes of polyethylene on a LaOCl spherical cap. The inset images show the correlated optical microscopy overview of the 2, 10 and 60 min ethylene polymerized $MgO/MgCl_2$ caps. The scale bars depict the size in microns.

3. Time-of-Flight Secondary Ion Mass Spectrometry

The mass spectra obtained from the imaged region are the convolution of the spherical caps and the Si(100) substrate. Therefore, to study if the charged mass fragments (called secondary ions) originating from the spherical caps, a region of interest (ROI) was drawn inside the analysis software on top of the spherical caps. The resulting mass spectra of both this spherical cap ROI and the inverse ROI that represents the Si(100) substrate, are given in Figure S8a as well as the insets that show the drawn ROIs. Additionally, the first few sputter seconds were typically disregarded from the analysis, as shown in Figure S8b, due to the presence of adsorbed carbon species from the air that are removed in these first few sputter cycles. The cut-off was based on reaching a stable intensity profile for the *m/z* species.

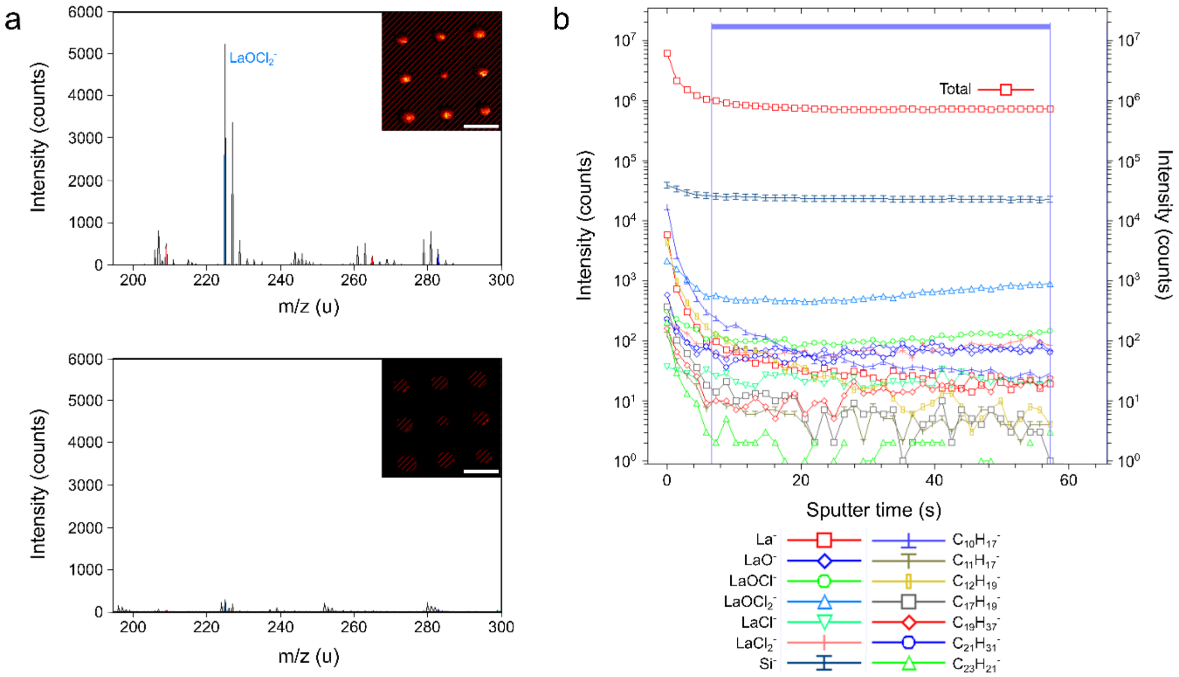

Figure S8. Time-of-Flight Secondary Ion Mass Spectrometry (ToF-SIMS) data and sputter profile explaining the chosen ROIs. (a) Mass spectra of the spherical cap model catalyst after 20 min polymerization time (inset map) recorded with ToF-SIMS. A ROI was picked selecting only the spherical caps, removing non-relevant mass fragments from the analysis. The ROI (top spectrum, all unmarked area in the inset map) contains the majority of the catalyst ($LaOCl_2^-$ indicated) and PE (repeating unit) compared to the substrate (bottom spectrum). (b) To avoid the interference of surface adsorbed species from the analysis, a z-ROI was chosen after the initial exponential increase/decrease of the organic fragments passed.

A selection of mass fragments is shown in Figure S9. Based on the ion generated SE images, the polyethylene phase can be clearly distinguished from the LaOCl spherical caps. It should be mentioned here that the ion counts for the $LaOCl^-$ fragment is typically orders of magnitude higher than for polyethylene fragments such as $C_9H_{13}^-$. This may be related to charging effects and the low ionisation probability of polyolefins, making them particularly challenging to be measured with ToF-SIMS. Additionally, the sample geometry (spherical caps with rough surfaces after polymerization instead of planar systems) could also cause adverse effects here. The $C_9H_{13}^-$ mass fragment was chosen here to represent the polyethylene phase as based on the work by Kern *et al.* [5]. This polyethylene mass fragment is also found for the pristine sample, but with considerably lower ion counts or intensity and is thus more likely to be from adventitious carbon due to air exposure of the samples. Nevertheless, as is mostly visible for the 2 and 10 min maps, the $C_9H_{13}^-$ mass charge fragment shows strong correlation with the polyethylene phase visible in the SE images.

The integrated intensity for this fragment, found in the different mass spectra, is plotted in Figure S10. As the polymerization time increases, more of this fragment is found (note that all measurement points have the same sputter time) until after 10 min a saturation effect starts to appear. This is in line with the AFM analysis performed in Figures S11-13, where the polyethylene volume on the external surface of the LaOCl spherical caps was estimated for different ethylene polymerization times and showed a saturation of the estimated polyethylene volume at intermediate polymerization times. This could be explained by a mass transfer limitation effect occurring at such intermediate polymerization times, where the fragmentation rate is not sufficiently high yet to facilitate high enough diffusion of ethylene monomers to the active sites within the spherical cap composite phase and hence a decline in the polymerization rate is observed.

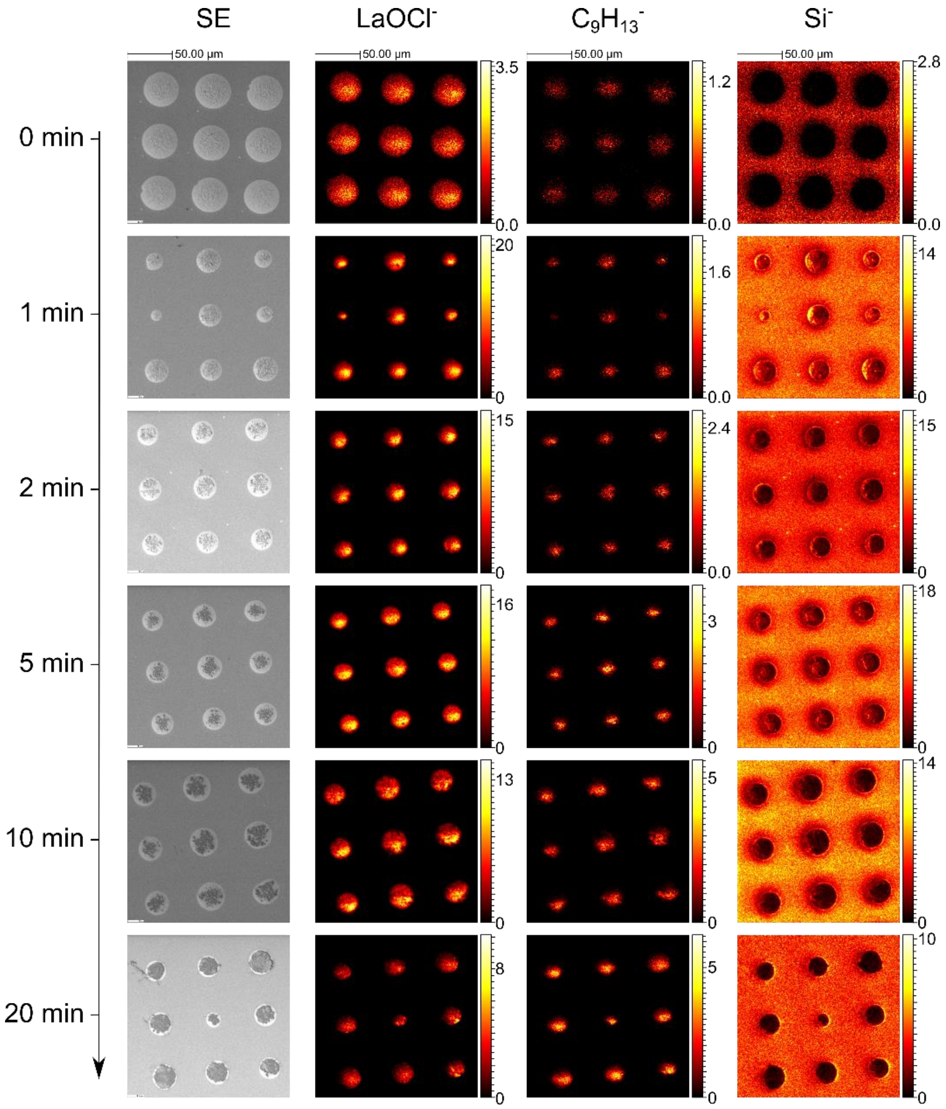

Figure S9. Time-of-flight secondary ion mass spectrometry (ToF-SIMS) secondary electron (SE) images, complemented by a selection of mass fragments, for different times of ethylene polymerization. The $LaOCl^-$ fragment is chosen to represent the polymerization catalyst, while the $C_9H_{13}^-$ fragment is representing the polyethylene. The Si^- fragment is shown to highlight the substrate on which the spherical caps were synthesized, and emphasizes that the LaOCl is only present in the patterned area as intended during the synthesis.

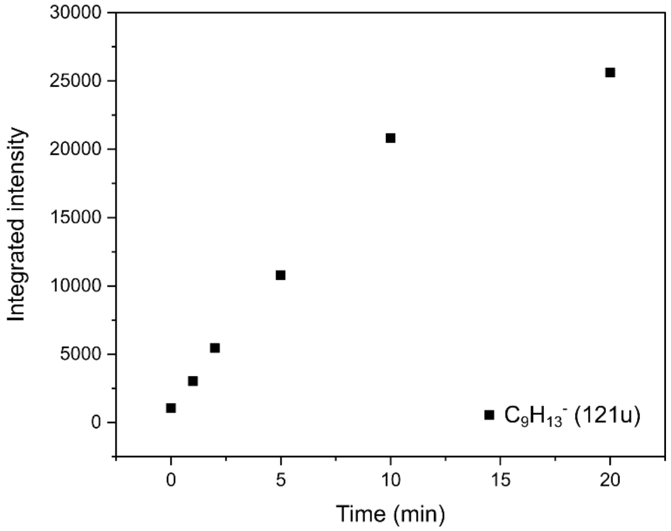

Figure S10. Integrated area of the $C_9H_{13}^-$ mass fragment. Representing polyethylene, the integrated area of the $C_9H_{13}^-$ mass fragment (m/z = 121 u), also shown in the mass images in Figure S9, is shown after increasing times of ethylene polymerization. Whereas first a linear increase is found, the integrated intensity starts to flatten out after roughly 20 min of ethylene polymerization. This indicates that the surface coverage of each island increases until a nearly full surface coverage is obtained.

4. Scanning Probe Microscopy

Atomic Force Microscopy (AFM) was used to investigate the topological and morphological evolution of the LaOCl spherical caps as a function of ethylene polymerization time. Figure S11 shows the topological images of 6 LaOCl spherical caps per ethylene polymerization time. The height contrast is set to be identical for the 0 to 20 min of polymerization time at a 0-1µm range. However, the ethylene polymerized LaOCl spherical caps of the 60 min sample were considerably thicker on average and therefore required a different height contrast as shown ranging from 0-4 µm. Purely based on the topological information provided by AFM it becomes clear that these LaOCl spherical caps become covered with polyethylene fibers of varying lengths already at ethylene polymerization times as early as 1 min. With an increase of polymerization times, thicker blobs of polyethylene start to appear (dark purple in color). It should be noted here that due to the different height scale bar in the 60 min sample, large features may appear to have a lighter (green-blue) color, which would have appeared dark purple with the height contrast used for the 0-20 min samples.

However, using purely the height of the polymer features itself isn't accurate enough to describe the polymer yield per polymerization time, as the patterns can vary in shape (diameter or height) even within the same wafer substrate. Instead, our approach to attempt to track the polymerization yield as a function of time is shown in Figure S12. The individual micrographs were first flattened on the background (substrate) and then *x* and *y* cross-sectional profiles were taken from the background through the center of the spherical caps. From the resulting line profiles, the height (*h*) and cap base radius (*a*) were measured by fitting the visible parts of uncovered catalyst with a power function. From these values the catalyst caps volumes were approximated with the following equation 3:

$$V = \pi . h \,(a^{\frac{1}{4}} + h^{\frac{1}{3}}) \qquad (3)$$

where V is the cap volume in µm^3, h is the height of the cap in µm and a is the cap base radius, or chord length, in µm [6]. It should be said here that this approach does not allow us the quantify the amount of polyethylene present within the spherical cap. For such an approach, X-ray nanotomography techniques, such as ptychography X-ray computed tomography as done in our previous works could offer a solution [7,8].

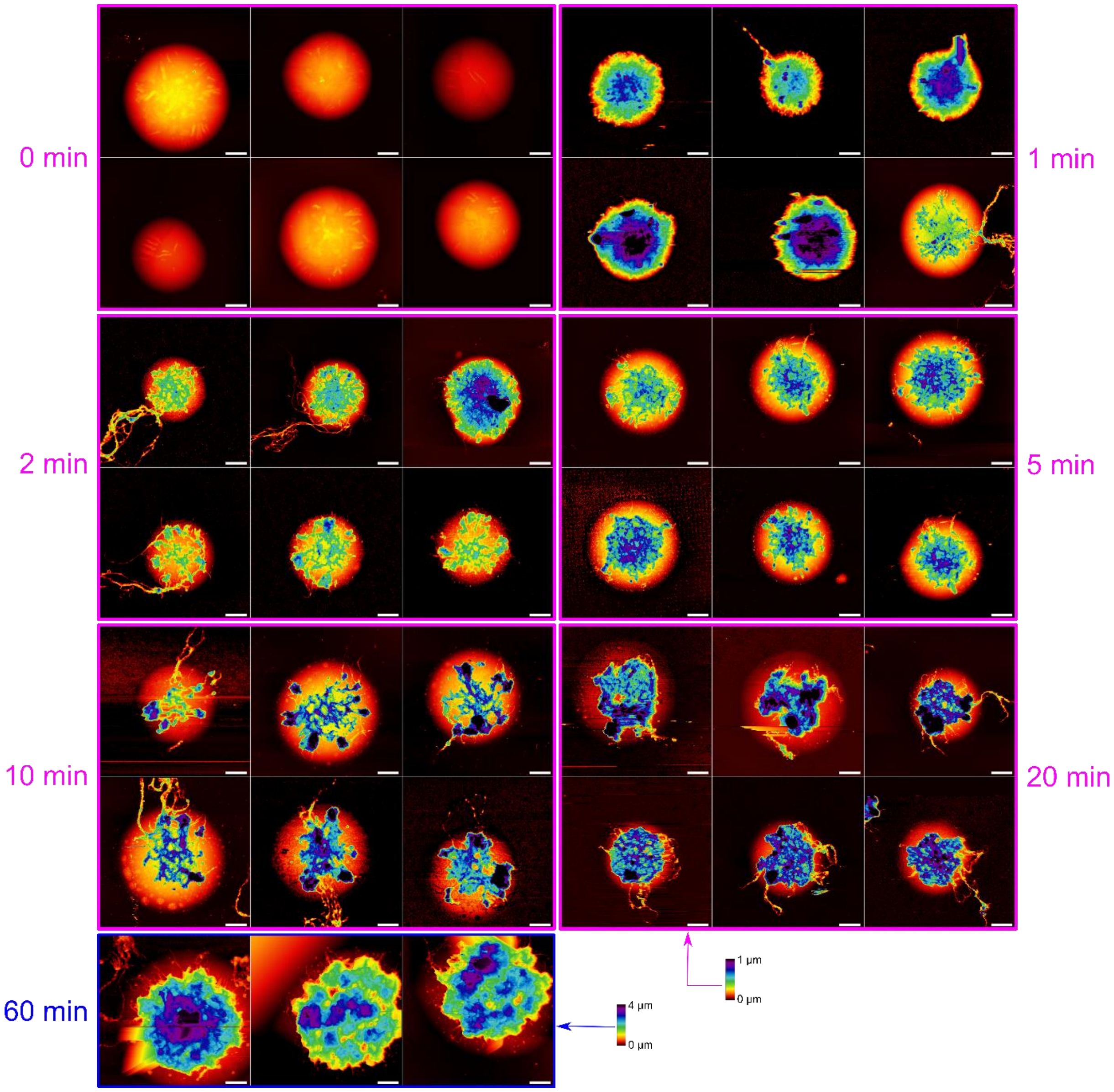

Figure S11. Atomic Force Microscopy (AFM) on individual catalyst caps after different ethylene polymerization times. Six LaOCl spherical caps were scanned per ethylene polymerization time, all shown using a z contrast of 1 µm, with the exception of 60 min, where only 3 viable micrographs could be obtained shown with a z contrast of 4 µm due to the excessive thickness of the polymer features which were too challenging for our scanning probe microscopy system. A general trend shows that the coverage and thickness of the formed polymer increase with increasing ethylene polymerization time. Furthermore, with the exception of 60 min, the edges of the patterned catalyst caps remain fairly visible whereas the centre part of the cap gets covered by PE strings and larger features. Notes: i) At 2 min, top-right; the catalyst consisted of a non-spherical shape. ii) 5 min, bottom-left; the catalyst was measured before by SEM analysis (explaining the grid-like background) iii) 10 min, top-left and bottom-right; some noise was found only on the background, not on the formed PE. White scale bars are 5 µm.

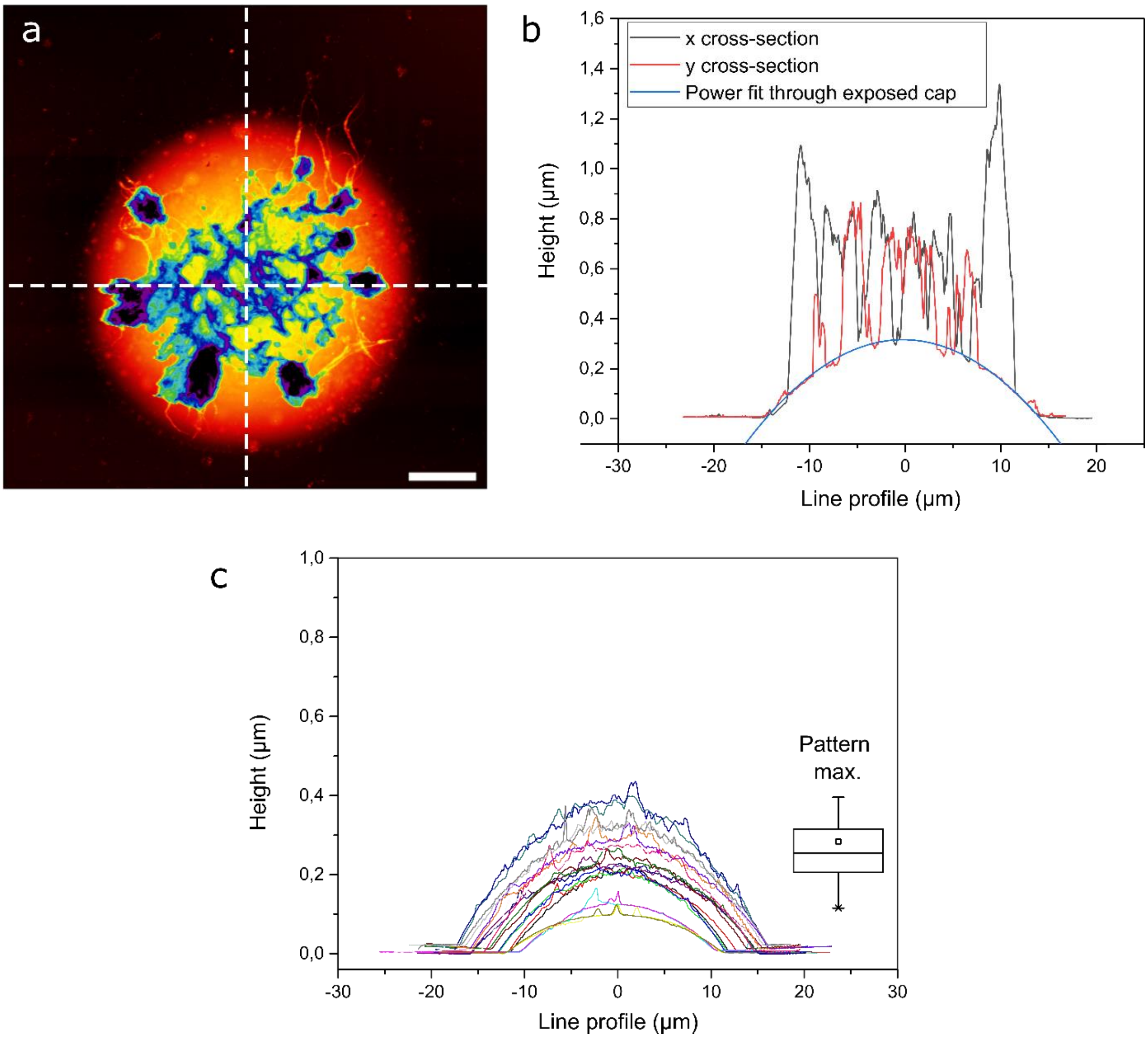

Figure S12. Estimation of the LaOCl spherical cap volume after ethylene polymerization and for the pristine hemispherical caps. A) The individual micrographs shown in Figure S11 were each flattened on their background (substrate) around the LaOCl spherical cap and polyethylene, shown here as example after 10 min polymerization (Figure S11, 10 min, center-top spot). B) Then, a *x* and *y* cross-section was taken and the resulting height profiles were fitted with a power function over the exposed polymer-free catalyst surface. Using the software measurement tools, the cap base radius or chord length and catalyst height were measured. These were then used to estimate the volume of the catalyst, which could subsequently be subtracted from the total volume obtained in the micrograph (above the background height) to result in a net volume of polyethylene. C) To show that the patterned catalyst indeed consist of hemispherical caps, not i.e. pancake- or disc shapes, and thus can be fitted with a power function, the cross section of all the measured pristine caps (for both Figure S11 and S13) are shown together with a box chart showing their maximum height.

The total volume of all features measured, which represents the LaOCl spherical cap and polyethylene phases, minus the estimated volume of the sole LaOCl spherical cap as described above, results in the estimated volume of polyethylene (per micrograph). This volume was plotted in Figure S13 for all micrographs recorded as shown in Figure S11, and for all full micrographs recorded with PiFM as shown in Figure S15 (*vide infra*), to visualize the increase of polyethylene over time ignoring the initial pore volume of the catalyst which might be filled up at the early stages.

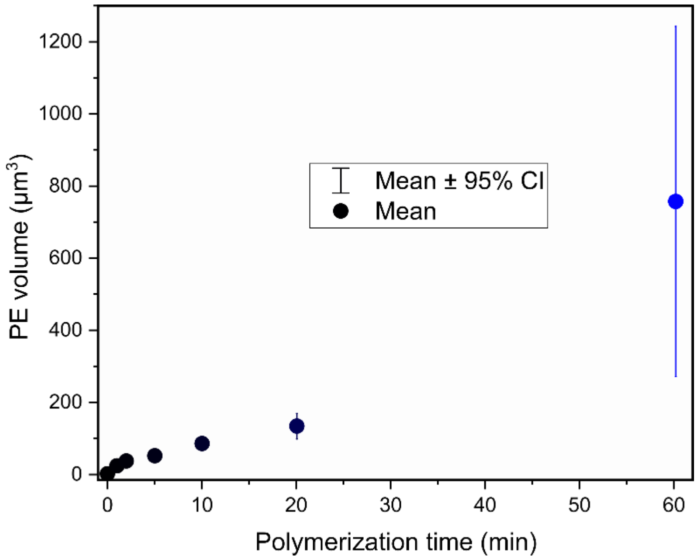

Figure S13. Mean volume of the polyethylene phase (PE) measured on the external LaOCl spherical caps as a function of ethylene polymerization time. The mean volume of the polyethylene phase growing on the external LaOCl spherical cap surface was calculated using the micrographs of Figure S11 and correction for the estimated volume of the LaOCl catalyst framework as discussed in Figure S12.

In Figure S14 the extreme sensitivity of the $MgO/MgCl_2$ caps to moisture is visualized with a time-laps series of AFM measurements. It is due to the typically rough shapes observed for these $MgO/MgCl_2$ samples that we have decided to refer to them simply as caps as they don’t resemble spherical caps as is the case for LaOCl. This seems to be caused by both the transformation step of $Mg(NO_3)_2$ x H_2O to MgO and the subsequent partial chlorination with $TiCl_4$ to $MgO/MgCl_2$. The first AFM measurement on the left was obtained with the use of an AFM operating inside a glovebox at <1 ppm O_2 and H_2O values whereas the subsequent two measurements were obtained with an AFM operating under ambient conditions after exposure times of respectively 15 and 35 min to moisture. All images were collected

with the same type of AFM apparatus and cantilevers as described in the methodology section. The line profile of each image obtained at the same X,Y positions shows a dramatic change in the morphology of the cap over time. Not only does the overall height of the cap collapse from almost 1.2 microns at the peak towards 0.8 microns, the minimum observed at a line profile position of 0 µm (X-axis of the plot) also changes. Interestingly, this value changes from almost 0 µm in height under controlled atmosphere to 0.4 µm in height after 15 min exposure and back to 0.2 µm in height after 35 min. This shows that the hydration and subsequent morphological changes induced on the $MgO/MgCl_2$ framework can be a rather slow process. Additionally, due to the severe morphological changes, care has to be taken especially when analyzing the pristine $MgO/MgCl_2$ caps or at low polymerization times when there is still a large exposed surface of $MgO/MgCl_2$.

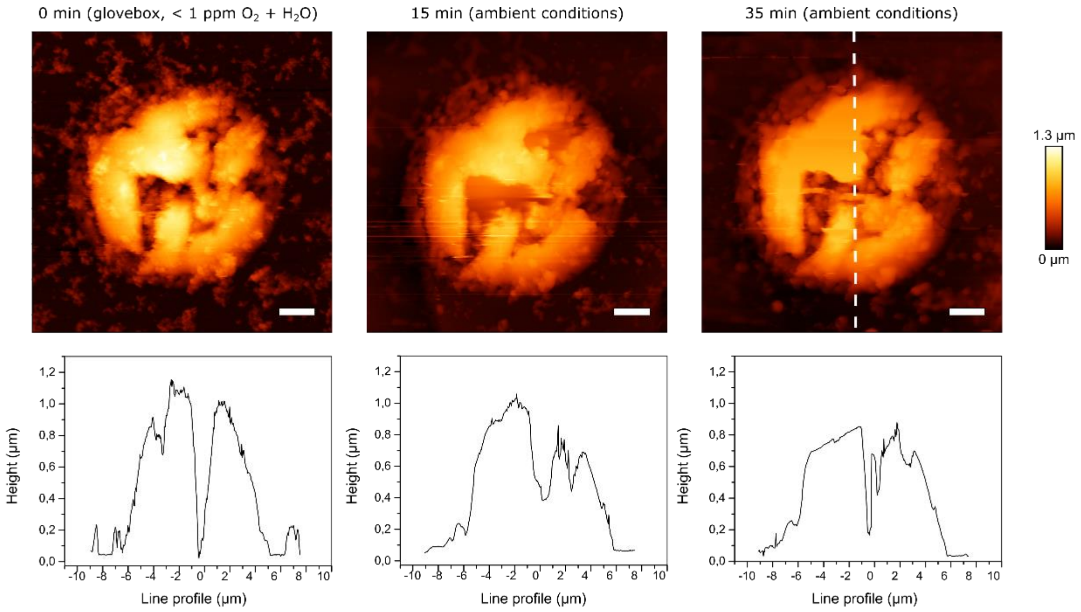

Figure S14. Time-laps AFM results on a single pristine $MgO/MgCl_2$ cap before (AFM positioned inside a glovebox operating at <1 ppm O_2 and H_2O) and after (AFM operating under ambient conditions) exposure to moisture. Additionally, on the bottom row the line profiles are provided going on the same position of each cap as shown via the dashed white line.

Going from 0-20 min ethylene polymerization a saturation effect of the polyethylene volume growing on the external surface of the LaOCl spherical cap is observed. This could be explained by first the formation of many extruded polyethylene fibres at early polymerization times as well as the filling of the internal pore network of the LaOCl spherical cap. As the internal pore network becomes filled with

a polyethylene phase that can't be assessed with this approach (since AFM only measured topological information and not internal), these extruded polyethylene fibres will show a non-linear decaying growth rate due to mass transfer limitations. This is because new ethylene monomers will now have to diffuse through a dense polyethylene phase within the internal pore network to reach the active sites. However, at 60 min of ethylene polymerization a drastic change is observed with a considerably higher average external polyethylene volume when compared to the 5-20 min samples. This sudden large increase of the 60 min sample corresponds well to the FIB-SEM observations that the internal LaOCl spherical cap has been completely disintegrated into small fragments dispersed well within the polyethylene phase, whereas for the 5-20 min samples most of the internal LaOCl framework was still intact, albeit with many crack lines and increasing amount of fragments observed. Therefore, the 60 min sample is expected to experience less mass transfer limitations due to the fragmentation of the framework, which is a necessary phenomenon to ensure polymerization activity [9,10].

In Figure S15 the micrographs recorded with PiFM are shown per PE time; 0 min shows a full spherical cap and the corresponding 1600 cm^{-1} signal, which most likely belongs to the C=O stretching vibration of surface absorbed carbonate species [11,12]. Interestingly, the 1600 cm^{-1} signal is enhanced at the surface cracks, most likely due to the presence of exposed unsaturated lattices or lattice defects that can chemisorb CO_2 and therefore also $TiCl_4$. It should be emphasized here that exposure to CO_2 only happens for the LaOCl spherical caps after either ethylene polymerization or in the case of this pristine sample when performing *ex-situ* surface analysis. Therefore, the presence of these carbonate absorbed species isn't expected to be present during the grafting of $TiCl_4$ in an inert atmosphere. The subsequent times show a full spherical cap (top left), zoom-in micrograph (top right) and the corresponding intensity maps for the 1461 + 1471 cm^{-1} vibration (magenta) and the 1600 cm^{-1} vibration (cyan), respectively.

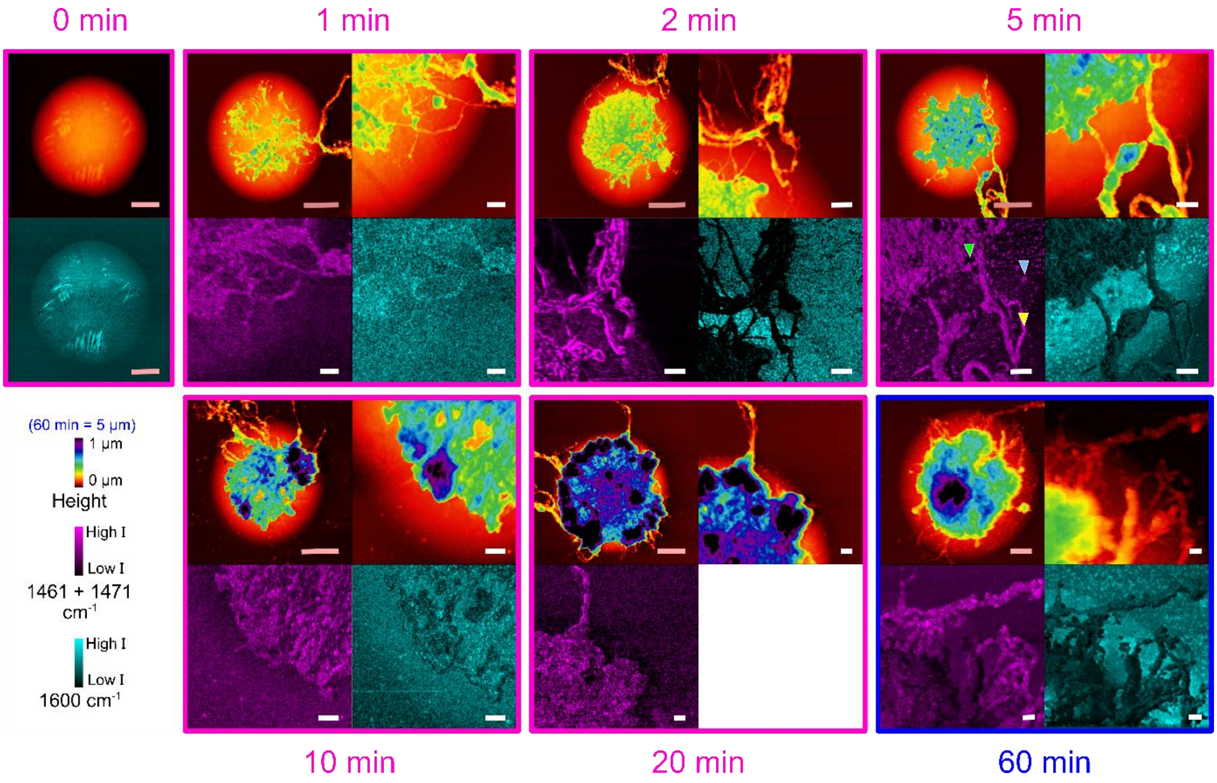

Figure S15. Individual Photo-induced Force micrographs of a full spherical cap, a zoom-in and the corresponding intensity maps for the 1461 + 1471 cm^{-1} and 1600 cm^{-1} vibrations on different ethylene polymerization times. Per polymerization time, the height micrographs are shown for a full spherical cap (top left) and a zoom-in (top right). The corresponding PiFM intensity maps are shown for the 1461 + 1471 cm^{-1} vibrations (magenta), and the 1600 cm^{-1} vibration (cyan) representing the PE and catalyst support, respectively. Note that the z-scale for all height micrographs is 1 µm, except for 60 min polymerization time, which has a z-scale of 5 µm. The pink inset scale bars represent 5 µm while the white scale bars represent 1 µm.

The extracted point spectra from the three markers, shown in the 1461+1471 cm^{-1} PiFM map in Figure S15 for the 5 min ethylene polymerized sample, are given in Figure S16. The markers show that on top of a polyethylene fibre that only the doublet peaks of the 1461+1471 cm^{-1} are present, which belong to a crystalline polyethylene phase. The other two markers on low intensity regions (of the 1461+1471 cm^{-1} signal) that are closer to respectively the centre and edge of the spherical cap show a broad band around 1600 cm^{-1}, which is attributed to adsorbed CO_2 surface species on the LaOCl framework after exposure of the sample to air and therefore allows us to also localize LaOCl with PiFM. On these positions, especially for the green marker one can still distinguish the doublet peak of polyethylene from the background, albeit with a poor signal to noise ratio.

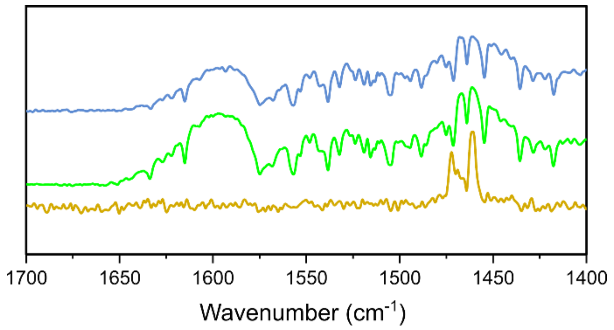

Figure S16. Point spectra recorded using Photo-induced Force Microscopy (PiFM) on the markers indicated in Figure S15 for the 5 min sample. Especially for the green and blue point spectra, a significant interference of water vapour can be observed for the LaOCl catalyst framework. The yellow spectrum is recorded on a polyethylene fibre and shows the characteristic polyethylene -CH_2- bending vibrations at 1461 and 1471 cm^{-1}.

The spectra shown in Figure 3c were obtained after averaging all spectra obtained on 9 different patterns per polymerization time. The spectra were pre-processed by applying a Whittaker baseline correction and normalization using the PLS Toolbox of Eigenvector. All individual spectra were then subjected to a multivariate curve resolution (MCR) analysis with non-negativity constraints, in which 4 spectral components were generated, as shown in Figure S17.

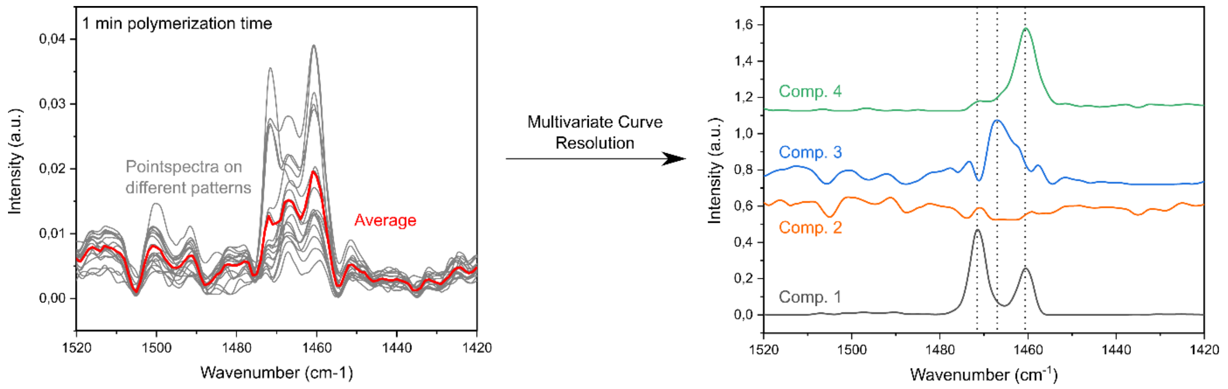

Figure S17. Point spectra recorded on 9 different LaOCl spherical caps after 1 min ethylene polymerization and the 4 different spectral components used in a multivariate curve resolution (MCR) analysis. The different spectral components represent the crystalline polyethylene phases (spectral components 1 and 4) as they consist purely of the -CH_2- bending vibrations of crystalline polyethylene in the orthorhombic phase, whereas spectral component 3 consists of a broad amorphous polyethylene -CH_2- bending vibration. Spectral component 2 represents interference caused by moisture present in the system and hence on the LaOCl surface and the PiFM scanning tip.

The recorded spectra were then fitted by summing the components multiplied by a certain score. Hence, this score represents the contribution of a certain component to this spectrum. Spectral components 1 and 4 represent the crystalline polyethylene -CH_2- bending vibrations, B_{1u} and B_{2u}, at respectively 1461 and 1471 cm^{-1}, whereas component 3 is comprised of a broad band centred around 1464 cm^{-1} and is attributed to an amorphous polyethylene phase [13]. It should be noted here that a 100% crystalline polyethylene phase isn’t feasible as the degree of crystalline is limited by the high viscosity of the polyethylene chains and thus this amorphous polyethylene contribution should always be present [14]. Spectral component 2 represents the water vapour interference, and hence we can compare the crystallinity within our own dataset by taking the percentage of the crystalline components (1 + 4) over all PE components (1 + 3 + 4), plotted in the main manuscript Figure 3c. Note that these percentages are an outcome of our MCR scores and hence don’t represent the mass or molar percentage of crystalline PE, but still can be used to study the evolution of crystallinity over time within this dataset.

Figure S18 shows the average PiF spectra recorded on multiple MgO/$MgCl_2$ caps after increasing polymerization times (topology micrographs of representative patterns are shown at the bottom). An identical MCR analysis with the same components was performed as done on the LaOCl system, and the resulting percentage of crystalline components over polymerization time is shown in Table S2 for both the MgO/$MgCl_2$ and LaOCl system (also shown in the main manuscript Figure 3c).

Noteworthy, we see a similar saturation of the fraction of crystalline components for both systems. However, the saturation for the Mg system seems to happen at lower ratios. This might be related to the difficulty experienced while measuring the MgO/$MgCl_2$ system: the caps were very irregular due to hydration under ambient conditions leading to frequent presence of PiFM tip artefacts that can influence the quality of the spectra obtained. This becomes clear in Table S2 with the larger error for the MgO/$MgCl_2$ system, especially at earlier polymerization times where less polyethylene is formed and more MgO/$MgCl_2$ is exposed. It should be noted that these percentages represent a ratio of MCR components and not an absolute degree of polyethylene crystallinity. Nonetheless, we can conclude that the saturation of the fraction of crystalline components takes place for both systems already after roughly 10 minutes of ethylene polymerization under mild slurry-phase conditions.

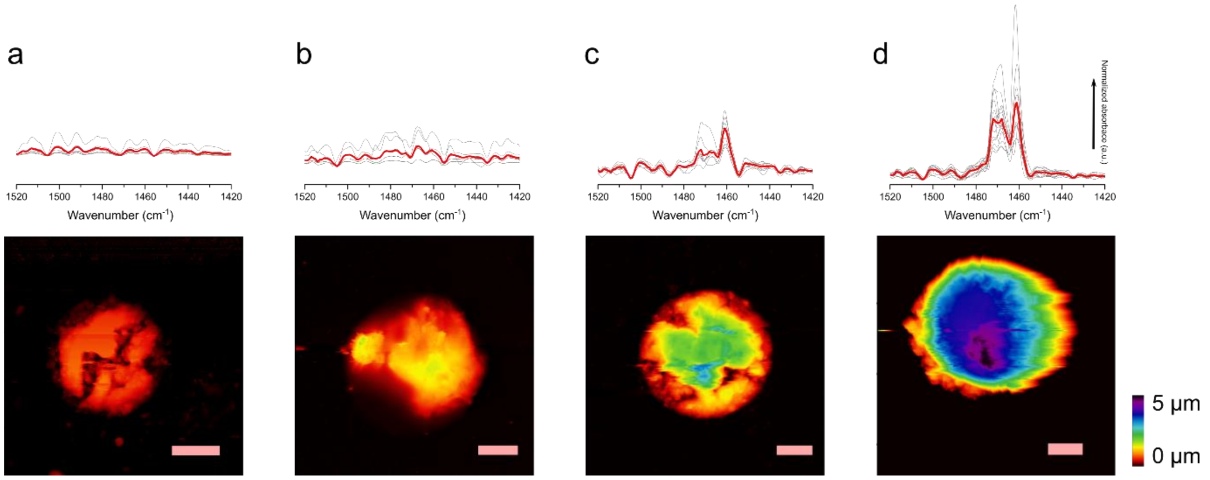

Figure S18. Photo-induced force microscopy (PiFM) point spectra (top) recorded on multiple (10-15) $MgO/MgCl_2$ caps after a) 0, b) 2, c) 10 and d) 60 min polymerization time. Atomic force microscopy images recorded using PiFM of representative patterns are shown at the bottom. The red spectrum represents the average of all recorded spectra (grey) for that specific polymerization time as also performed in Figure S17 for the LaOCl spherical cap samples. The scale bars represent 2 µm.

Table S2. Overview of the fraction of crystalline components (1461 cm^{-1} and 1471 cm^{-1} bands) versus the amorphous single amorphous component (broad band at 1463 cm^{-1}). The fractions were assessed with MCR analysis on the PiF spectra (as explained in Figure S17) obtained at respectively, 2, 10 and 60 min of ethylene polymerization on both $MgO/MgCl_2$ caps and LaOCl spherical caps after functionalization with $TiCl_4$.

**Sample**	**Polymerization time (min)**		
	**2**	**10**	**60**
**$MgO/MgCl_2$**<br>***Mean of crystalline MCR components (%)***	39,17 ± 10,4	64,4 ± 5,8	62,5 ± 6,0
**LaOCl**<br>***Mean of crystalline MCR components (%)***	49,4 ± 4,8	88,6 ± 4,5	80,2 ± 4,4

5. Scanning Electron Microscopy

In Figure S19, an overview is given of the SEM results of all ethylene polymerization times, starting at the top with a pristine LaOCl spherical cap sample up to 60 min of ethylene polymerization at the bottom. From left to right first, an overview of multiple LaOCl spherical caps at a specific ethylene polymerization is provided, followed by a single spherical cap and finally a zoom-in on this spherical cap. Starting at the 1 min ethylene polymerized sample, it becomes clear that the polyethylene fibres that are extruded out of the spherical caps contain large amounts of small LaOCl fragments. The extrusion of the polyolefin fibres has been reported for immobilized single-site catalysts in well-defined microporous and mesoporous support matrixes for polyolefin catalysts, such as the silica-based MCM-41 and SBA-15 with pore diameters in the range of 2-25 nm [15-20]. The small LaOCl fragments must have been peeled off the internal and possibly external surface of the catalyst. This indicates the presence of the shrinking core or also called layer-by-layer fragmentation model, as illustrated in Figure S21. After ten min of ethylene polymerization, the external surface of the centre of the spherical caps are observed to be fully covered in a polyethylene fibre network. Additionally, starting at this polymerization time, large LaOCl fragments are observed to lay intertwined with and on top of this polyethylene fibre network. The zoom-in on the 10 min ethylene polymerized sample shows one such large LaOCl fragment laying on top of the centre of the spherical cap and is actually observed to already have been cleaved into several smaller fragments that are being pushed away from each other by the growing polyethylene fibres. This internal cleavage shows the presence of the continuous bisection fragmentation model, as illustrated in Figure S21, occurring even at the external surface of the spherical cap.

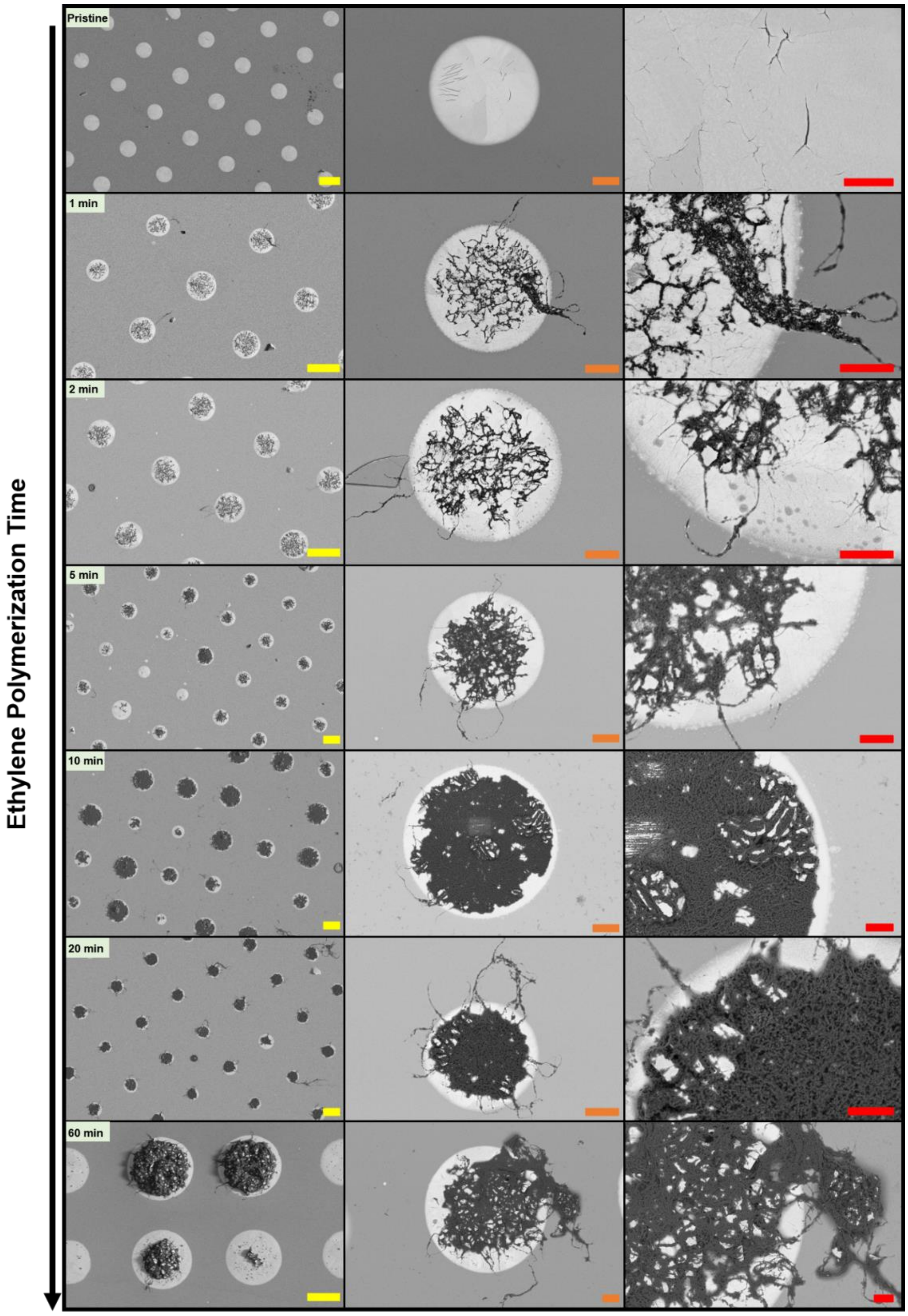

Figure S19. Secondary Electron Microscopy (SEM) results on the LaOCl spherical caps after different ethylene polymerization times. From left to right first, an overview consisting of multiple LaOCl spherical caps at a certain polymerization time are given followed by a single spherical cap and finally a zoom-in on that specific spherical cap. From top to bottom, an increase of ethylene polymerization time is given, starting at a pristine non-polymerized sample and going up to 60 min of ethylene polymerization. The yellow, orange and red scalebars represent a width of respectively 20, 5 and 2 microns.

In Figure S20 an overview is given of the SEM results on the reference $MgO/MgCl_2$ caps for a pristine, 2, 10 and 60 min ethylene polymerized samples. From left to right, full single caps are first shown in a top-down fashion followed by a zoom-in and then repeated as cross-sectional images on the exact same caps with again a full size image and a zoom-in. The pristine $MgO/MgCl_2$ caps, which have been exposed to moisture due to the absence of a transfer chamber for the SEM, shows a rough appearing surface with some large exposed cracks and seems to bleed onto the Si(100) substrate. This bleeding effect could be due to the rapid vacuum treatment used to evacuate the SEM chamber upon sample transfer of the hydrated $MgCl_2$ layer. This high moisture sensitivity of the $MgO/MgCl_2$ caps was also observed earlier in supplementary information section 4 with AFM time-lapse measurements before and after exposure of the $MgO/MgCl_2$ pristine caps to moisture. Upon 2 min of ethylene polymerization the first polyethylene fibers and globules can be observed on the caps, albeit that the weak Z-contrast between $MgO/MgCl_2$ and polyethylene as compared to LaOCl make it more difficult to observe at this stage. It is only upon increased polymerization times, ergo 10 and 60 min, that the imaging contrast between the $MgO/MgCl_2$ support matrix and the formed polyethylene becomes clear enough due to the strong fragmentation of the original support matrix to spatially resolve the different chemical phase. Here it becomes clear that the $MgO/MgCl_2$ support matrix is mainly fragmenting internally into successively smaller units according to the continuous bisection. Especially for the 60 min polymerized sample, a complete disintegration of the framework is observed where small $MgO/MgCl_2$ fragments only a few tens of nm in size are homogeneously distributed within the formed polyethylene phase. At the highest point in this specific cross-section, a thickness of 7.2 µm is obtained, compared to the 5.7 µm thickness for the 60 min LaOCl spherical cap. It should be mentioned here that more representative values would be obtained with quantitative 3-D or topological measurements such as AFM (albeit that this sample is too thick for the height limits of the AFM piezo stage) or X-ray nano-tomography.

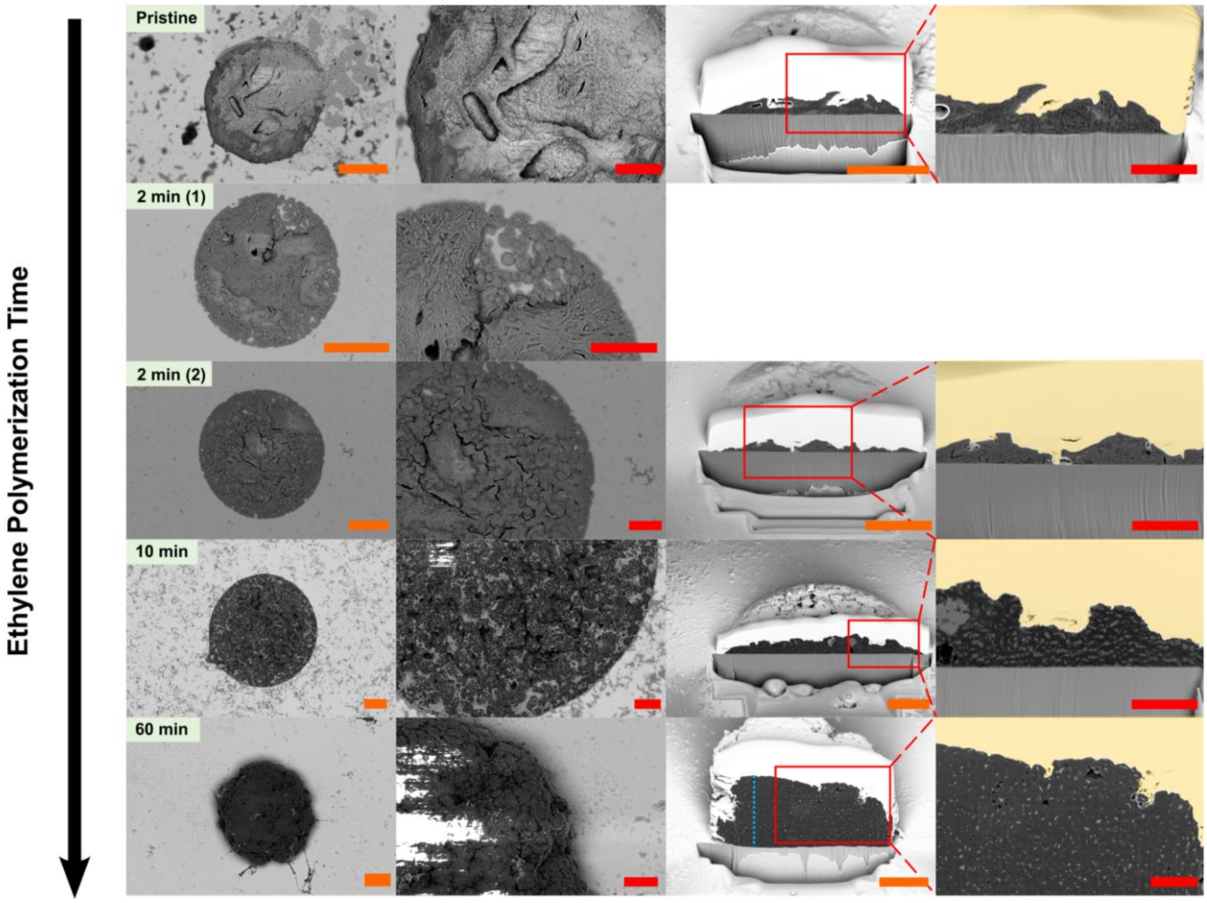

Figure S20. Secondary Electron Microscopy (SEM) results on the reference $MgO/MgCl_2$ caps after different ethylene polymerization times. From left to right first a top-down view of a single cap is shown at a certain polymerization time followed by a zoom-in on that specific cap. This is followed by cross-sectional images of the same caps and the respective zoom-ins based on the red box. From top to bottom, an increase of ethylene polymerization time is given, starting at a pristine non-polymerized sample and going up to 60 min of ethylene polymerization. The orange and red scalebars represent a width of respectively 5 and 2 microns. The cyan dashed line in the cross-sectional SEM image of the 60 min ethylene polymerized $MgO/MgCl_2$ cap depicts a length of 7.2 microns.

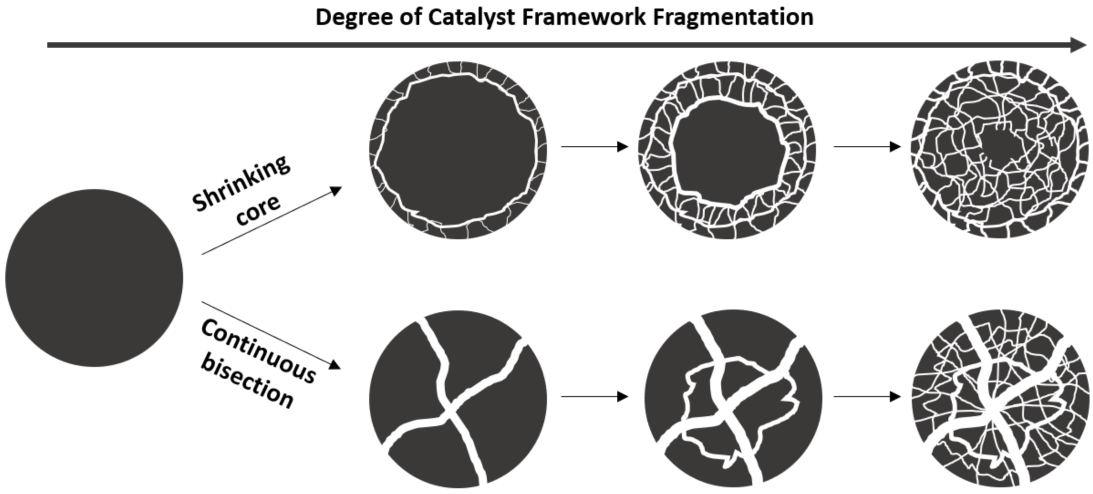

Figure S21. Schematic representation of the shrinking core and continuous bisection limiting modes of catalyst framework fragmentation. The shrinking core fragmentation mode starts at the surface of the catalyst particle whereas the continuous bisection fragmentation mode starts at the core outwards.

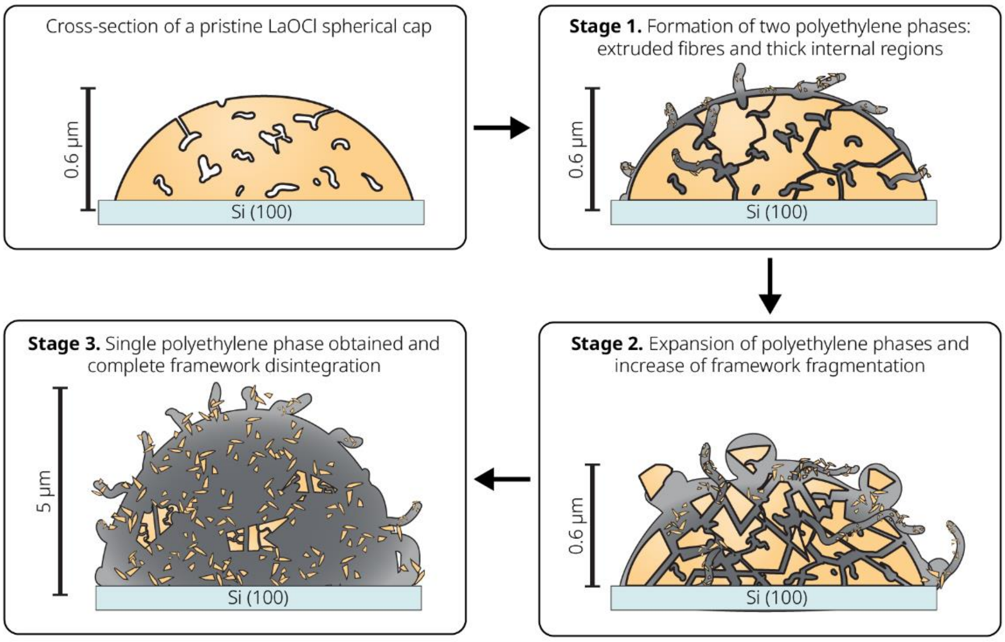

Figure S22. A schematic representation of the fragmentation behaviour of the LaOCl spherical cap model system during ethylene polymerization. Three different polymerization stages are drawn, starting from the pristine spherical cap. In the first stage (i) the internal macroporous cavities and externally accessible cracks of the LaOCl spherical cap starts to be filled with polyethylene and leads to both the extrusion of polyethylene fibres from these cracks, as well as a locally heavy degree of framework fragmentation at the outer rim of the spherical caps, indicating the occurrence of both the shrinking

core and continuous bisection fragmentation models in Figure S21. In the second stage (ii) both polymer phases, that is the extruded polyethylene fibres and the thick internal polyethylene regions at the outer rims of the spherical caps, keep growing steadily and cause the formation of additional cracking lines on the framework. Finally, in the third stage (iii) the original morphology of the spherical caps has disintegrated into both large and small LaOCl fragments that are dispersed uniformly throughout the polymer phase, showing that the continuous bisection fragmentation model has become dominant.

The SEM images and EDX maps, shown in Figure S23 of the pristine and 20 min ethylene polymerized sample, are of the same spherical caps, as shown in Figures 2 and 4. EDX shows the presence of La, O and Cl, the constituents of LaOCl both before and after ethylene polymerization. The high photon energies of especially La at 4651 eV for the $L\alpha_1$ emission line means that the formed polyethylene phase (carbon binding energy is ~284 eV) will barely lead to attenuation of these photons and therefore cause the La phase to be easily observable through the polymer phase. For the pristine LaOCl spherical cap, only a small contribution of carbon is found, most likely due to the adsorption of carbonate species due to exposure to air during sample transfer. However, after 20 min of ethylene polymerization, the carbon signal of the formed polyethylene phase becomes clear.

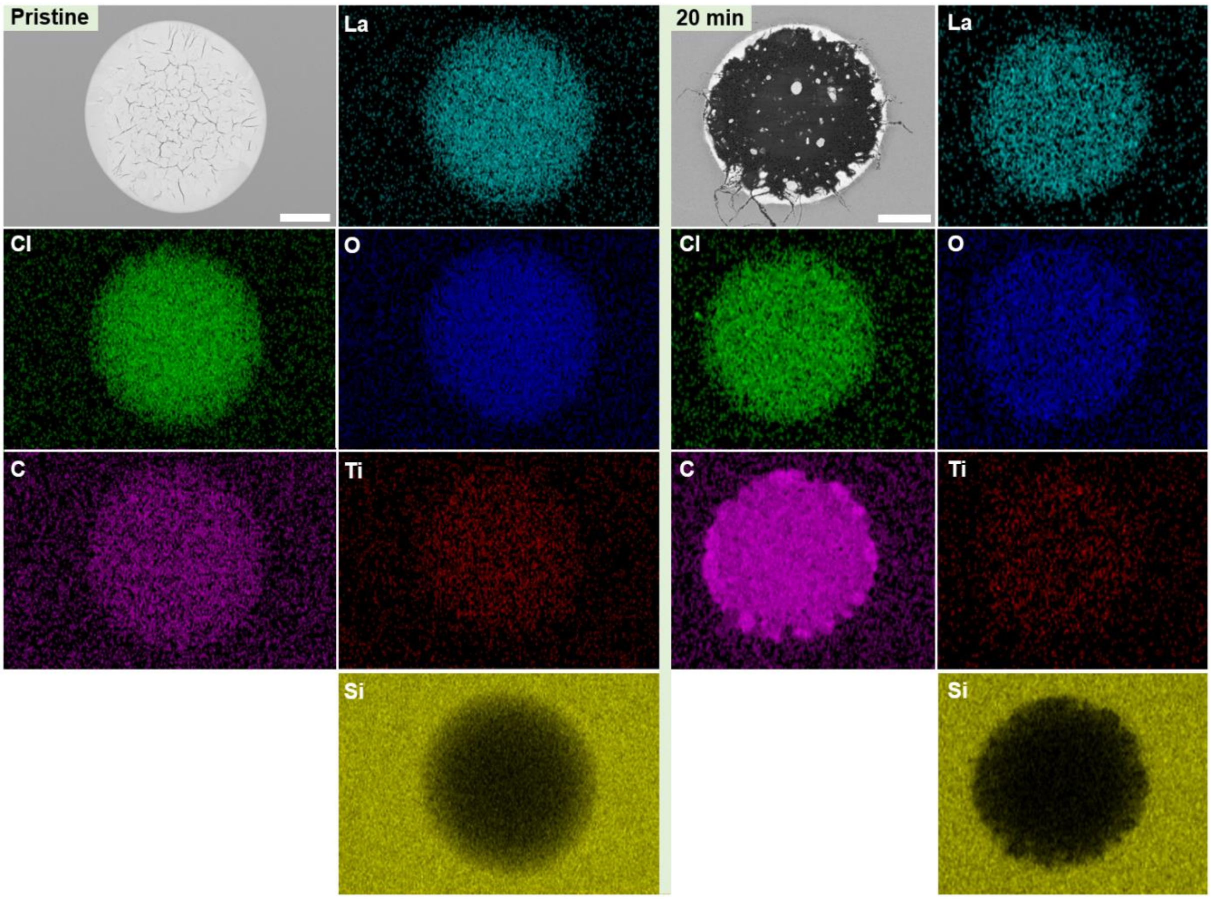

Figure S23. Top-view Secondary Electron Microscopy Energy Dispersive X-ray spectroscopy (SEM-EDX) results on the LaOCl spherical caps before and after ethylene polymerization. On the left and right side, the top-view back-scattered electron images of respectively the pristine and 20 min ethylene polymerized spherical caps are given alongside the EDX maps of La, Cl, O, C, Ti and Si. The scalebars depict a width of 10 microns.

The SEM and EDX images, shown in Figure S24, belong to the cross-sectional regions of the pristine, 2 min and 60 min of ethylene polymerization, as shown in Figure 4. The cross-sections are taken around the centre of each spherical cap. For the pristine LaOCl spherical cap, no carbon phase is observed within the cross-section, as expected, whereas for the 2 min ethylene polymerized sample a faint carbon signal can be seen, albeit difficult due to the deposition of carbon on the platinum coating above the spherical cap during the sputtering process. For the 60 min ethylene polymerization, a clear carbon phase can be observed now, as well as the LaOCl fragments dispersed within the carbon phase.

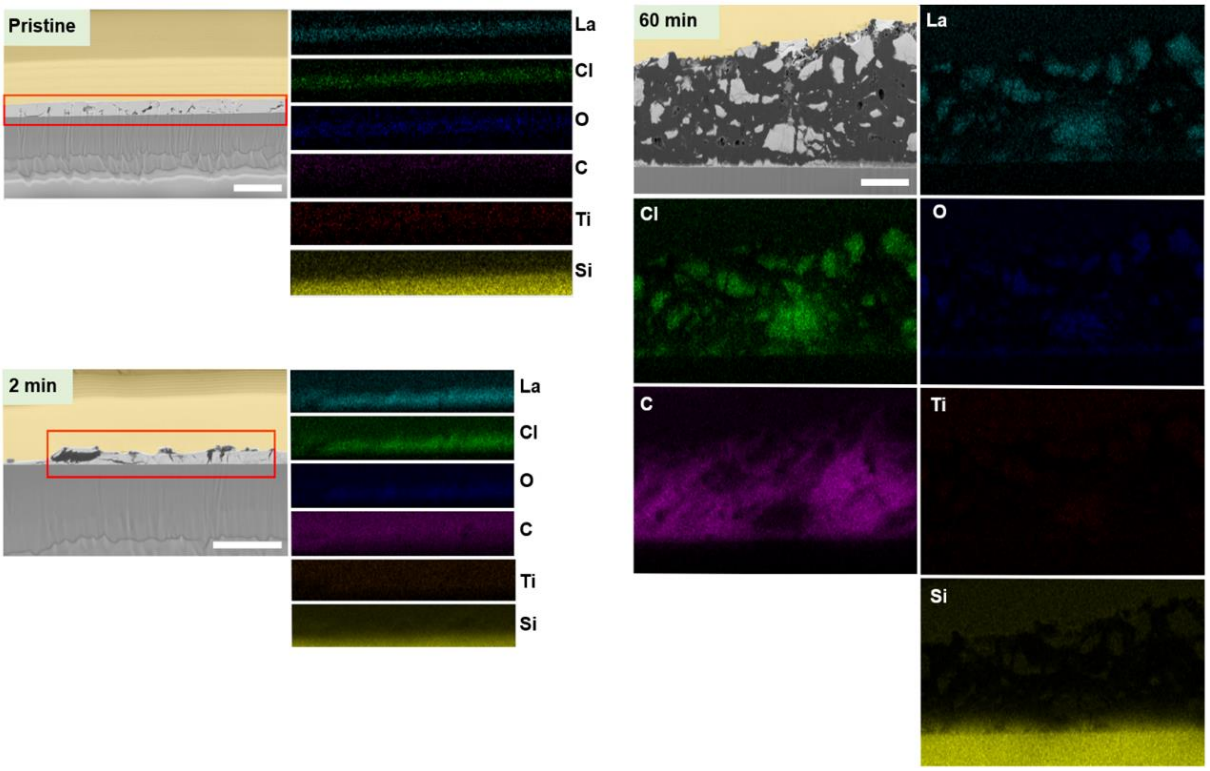

Figure S24. Cross-sectional Secondary Electron Microscopy Energy Dispersive X-ray spectroscopy (SEM-EDX) results on the LaOCl spherical caps before and after ethylene polymerization. The cross-sectional back-scattered electron images alongside the EDX maps of La, Cl, O, C, Ti and Si are given for the pristine, 2 min and 60 min of ethylene polymerization. The red boxes in the SEM images portray the area of the EDX maps in case it

is smaller than the SEM image shown. The scalebars depict a width of 2 microns. A yellow, transparent overlay is provided that indicates the coated Pt layer, all though in some cases it is observed to penetrate into the porous polyethylene layer.

In Figure S25, the SEM-EDX images are shown of the pristine and 60 min ethylene polymerized reference $MgO/MgCl_2$ caps from a top-down perspective. The SEM-EDX of the pristine sample shows the presence of the Mg, O and Cl signals, which is expected from the $MgO/MgCl_2$ core-shell structure as reported by Chammingkwan *et al.* [21]. Despite the use of $TiCl_4$ as the chlorinating agent to convert MgO partially into $MgO/MgCl_2$ as well as that $TiCl_4$ is the pre-active site, EDX shows no clear signal for Ti on the pristine sample. This might indicate a lower weight loading of Ti on the $MgO/MgCl_2$ caps with respect to the LaOCl spherical caps where the Ti signal was noticeable in Figure S23. The odd shape of the pristine $MgO/MgCl_2$ cap could be due to the hydration of the framework upon transfer through ambient conditions towards the SEM apparatus, who's severe effect was shown before by the AFM time-laps in Figure S14. The SEM-EDX images of the 60 min sample clearly show the presence of a highly intense carbon signal, which is attributed to the polyethylene phase when taking the Raman and PiFM results into account. Here the carbon signal is so intense that no clear presence of the Mg, O or Cl peaks from the catalyst framework can be observed.

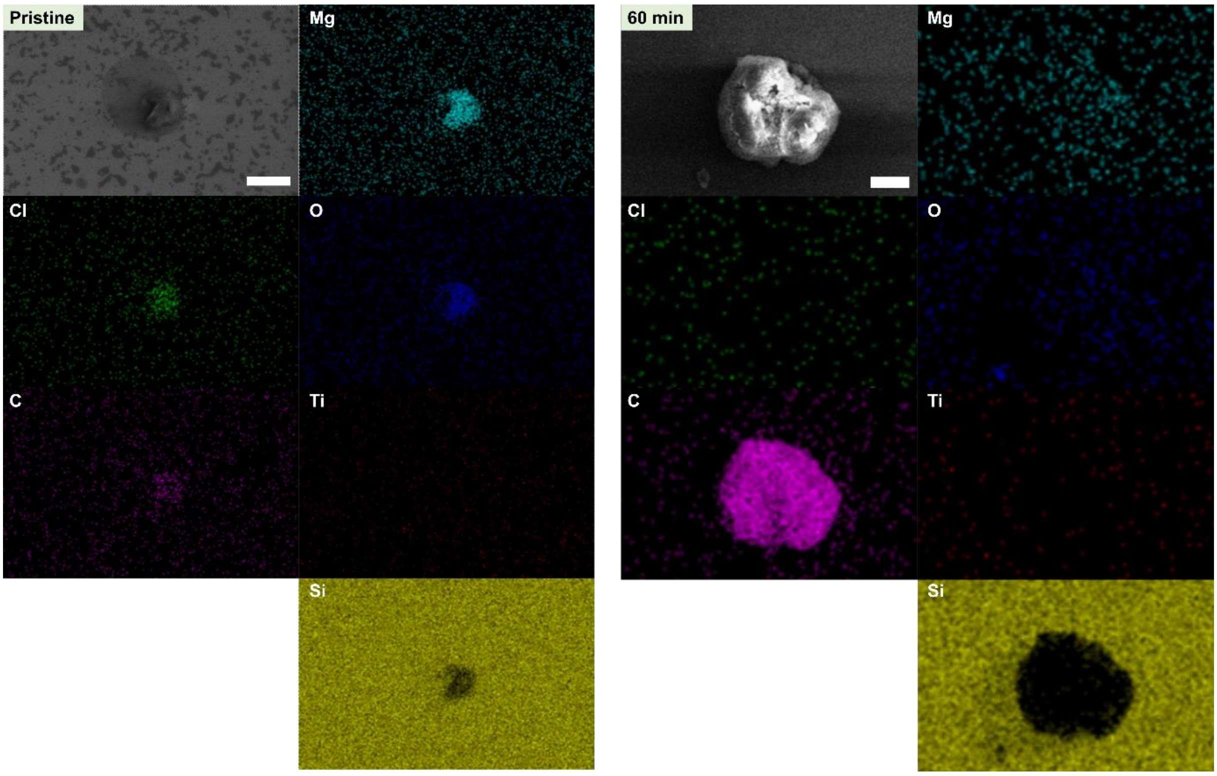

Figure S25. Top-view Secondary Electron Microscopy Energy Dispersive X-ray spectroscopy (SEM-EDX) results on the reference $MgO/MgCl_2$ caps before and after ethylene polymerization. On the left and right side, the top-view back-scattered electron images of respectively the pristine and 60 min ethylene polymerized caps are given alongside the EDX maps of Mg, Cl, O, C, Ti and Si. The scalebars depict a width of 10 microns.

In Figure S26, the cross-sectional SEM-EDX images are shown for the pristine, 2 and 60 min ethylene polymerized reference $MgO/MgCl_2$ caps. The pristine sample shows that the extent of MgO chlorination seems to be uniform from the external surface towards the interface with the Si(100) substrate and therefore $TiCl_4$ pre-active sites could potentially speaking be chemisorbed also uniformly throughout the $MgO/MgCl_2$ volume (on exposed, unsaturated lattices that is) all though again the Ti weight loading seems to be below the detection limit of these EDX experiments. For the 2 min ethylene polymerized sample, the Mg, Cl, O and C signals are all visible whereas for the 60 min ethylene polymerized sample, Mg is barely visible with Cl and O signals being too diluted within the highly intense C signal from the formed polyethylene. The zoom-in cross-sectional image of this same 60 min ethylene polymerized cap in Figure S20 also showed how finely the original $MgO/MgCl_2$ has fragmented as compared to the 60 min ethylene polymerized LaOCl spherical cap where larger support fragments were still clearly visible with EDX.

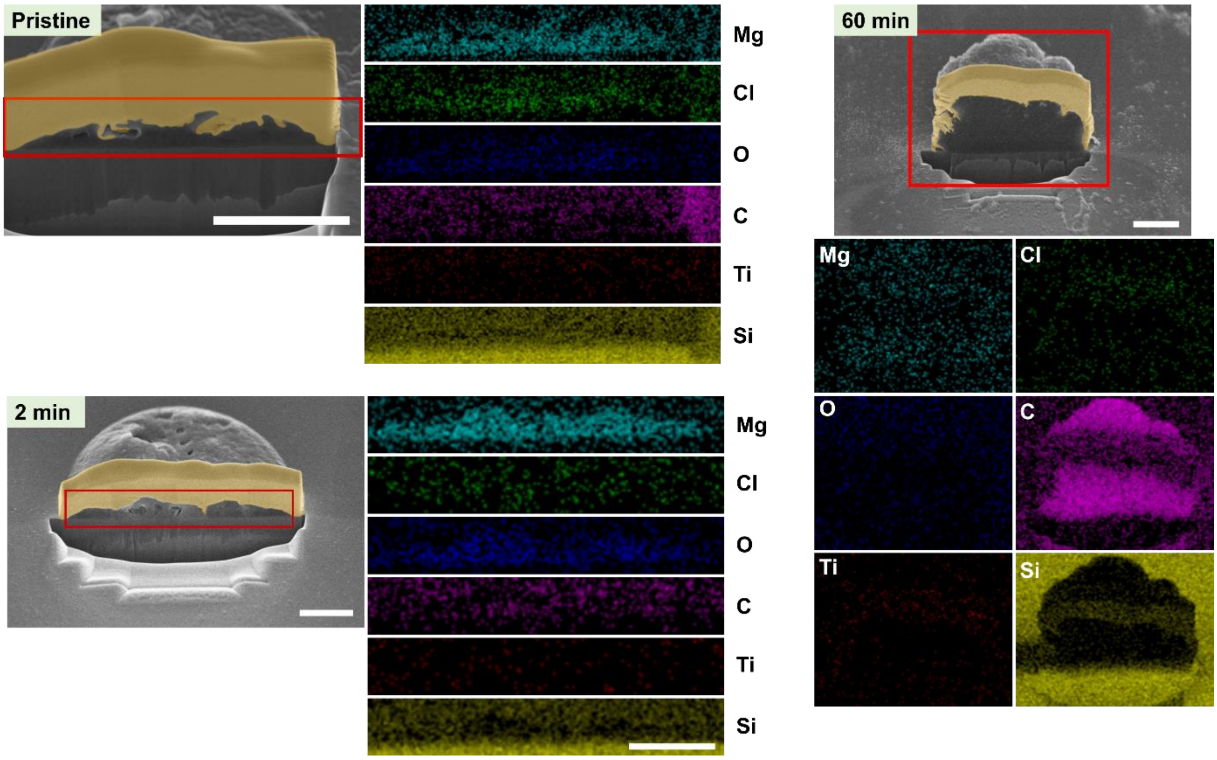

Figure S26. Cross-sectional Secondary Electron Microscopy Energy Dispersive X-ray spectroscopy (SEM-EDX) results on the refence $MgO/MgCl_2$ caps before and after ethylene polymerization. The cross-sectional back-scattered electron images alongside the EDX maps of Mg, Cl, O, C, Ti and Si are given for the pristine, 2 min and 60 min of ethylene polymerization. The red boxes in the SEM images portray the area of the EDX maps in case it is smaller than the SEM image shown. The scalebars depict a width of 5 microns. A yellow, transparent overlay is provided that indicates the coated Pt layer, all though in some cases it is observed to penetrate into the porous polyethylene layer.

Supplementary References

(1) Li, J.P.H., *et al.,* Understanding of binding energy calibration in XPS of lanthanum oxide by *in situ* treatment, *Phys. Chem. Chem. Phys.*, **21**, 22351-22358 (2019).
(2) Magni, E., Somorjai, G.A., Surface science study of model Ziegler-Natta catalysts, *Surf. Sci.*, **377**, 824-827 (1997).
(3) Van der Heijden, A.W.A.M., *et al.,* Destructive Adsorption of CCl_4 over Lanthanum-Based Solids: Linking Activity to Acid-Base Properties, *J. Phys. Chem. B.,* **109**, 23993-24001 (2005).
(4) Podkolzin, S.G., Manoilova, O.V., Weckhuysen, B.M., Relative Activity of La_2O_3, LaOCl, and $LaCl_3$ in Reaction with CCl_4 Studies with Infrared Spectroscopy and Density Functional Theory Calculations, *J. Phys. Chem. B,* **109**, 11634-11642 (2005)
(5) Kern, S., Kern, C., Rohnke, M., Mass spectra database of polymers for bismuth-cluster ToF-SIMS, *Surf. Sci. Spectra,* **26**, 025003 (2019).
(6) Oberg, E., Jones, F.D., Horton, H.L., Ryffel, H.H., *Machinery's handbook: a reference book for the mechanical engineer, designer, manufacturing engineer, draftsman, toolmaker, and machinist* (Industrial Press, New York, 1988).
(7) Bossers, K.W., Valadian, R., Zanoni, S., Smeets, R., Friederichs, Garrevoet, J., Meirer, F., Weckhuysen, B.M., Correlated X-ray Ptychography and Fluorescence Nano-Tomography on the Fragmentation Behavior of an Individual Catalyst Particle during the Early Stages of Olefin Polymerization, *J. Am. Chem. Soc.,* **142**, 3691-3695 (2020).
(8) Bossers, K.W., Valadian, R., *et al.,* Heterogeneity in the Fragmentation of Ziegler Catalyst Particles during Ethylene Polymerization Quantified by X-ray Nanotomography, *JACS Au*, 1, 852-864.
(9) Horácková, B., Grof, Z., Kosek, J., Dynamics of fragmentation of catalyst carries in catalytic polymerization of olefins, *Chem. Eng. Sci.*, (**62**, 5264-5270, 2007).
(10) Seda, L., Zubov, A., Bobak, M., Kosek, J., Kantzas, A., Transport and Reaction Characteristics of Reconstructed Polyolefin Particles, *Macromol. React. Eng.*, (**2**, 496-512, 2008).
(11) Manoilova, O.V. *et al.,* Surface Acidity and Basicity of La_2O_3, LaOCl, and $LaCl_3$ Characterized by IR Spectroscopy, TPD, and DFT Calculations, *J. Phys. Chem. B.,* **108**, 15770-15781 (2004).
(12) Alizadeh, S., Mousavi-Kamazani, M., Salavati-Niasari, M., Hydrothermal Synthesis of Rod-Like LaOCl Nanoparticles from New Precursors, *J. Clust. Sci.*, **26**, 645-652 (2015).
(13) Raff, R.A.V., Doak, K.W. *Crystalline Olefin Polymers Part 1*, (John Wiley and Sons Inc., New York, 1965).
(14) Whiteley, K.S., Heggs, T.G., Koch, H., Mawer, R.L., Immel, W., Polyolefins. In: *Ullmann's Encyclopedia of Industrial Chemistry* (Wiley-VCH, Weinheim, 2005).
(15) Ko, Y.S., Woo, S.I., Tailor-made Polymers: Via Immobilization of Alpha-Olefin Polymerization Catalysts Ch.10, (Wiley-VCH, Weinheim, 2008).
(16) Ko, Y.S., Han, T.K., Park, J.W., Woo, S.I., Propene polymerization catalyzed over MCM-41 and VPI-5 supported $Et(ind)_2ZrCl_2$ catalysts, *Macromol. Rapid Commun.,* **17**, 749-758 (1996).
(17) Kageyama, K., Tamazawa, J., Aida, T., Extrusion polymerization: catalyzed synthesis of crystalline linear polyethylene nanofibers within a mesoporous silica, *Science*, **285**, 2113-2115 (1999).
(18) Ye, Z., Zu, S., Wang, W., Alsyouri, H., Lin, Y.S., Morphological and mechanical properties of nascent polyethylene fibers produced via ethylene extrusion polymerization with a metallocene catalyst supported on MCM-41 particles, *J. Polym. Sci. Part B: Polym. Phys.*, **41**, 2433-2443 (2003).
(19) Dong, X., *et al.*, Preparation of nano-polyethylene fibers and floccules using MCM-41-supported metallocene catalytic system under atmospheric pressure, *Eur. Polym. J.,* **41**, 797-803 (2005).
(20) Kumkaew, P., Wanke, S.E., Parserthadam, P., Danumah, C., Kaliaguine, S., Gas-phase ethylene polymerization using zirconocene supported on mesoporous molecular sieves, *J. Appl. Polym. Sci.,* **87**, 1161-1177 (2003).
(21) Chammingkwan, P., Than, V.Q., Terano, M., Taniike, T., $MgO/MgCl_2/TiCl_4$ Core-Shell Catalyst for Establishing Structure-Performance Relationship in Ziegler-Natta Olefin Polymerization, *Topics Catal.,* **57**, 911-917 (2014).
